# Axially coordinated single-atom interface mitigates isolated K toward highly reversible anode-free K metal batteries

**DOI:** 10.1126/sciadv.aef1038

**Published:** 2026-07-24

**Authors:** Qian Liu, Meng Tian, Xueyu Lian, Zixiang Meng, Lin Zeng, Yongbiao Mu, Le Yu, Jingyu Sun

**Affiliations:** ^1^ College of Energy, Soochow Institute for Energy and Materials Innovations, Jiangsu Provincial Key Laboratory for Advanced Carbon Materials and Wearable Energy Technologies, Soochow University, Suzhou, China.; ^2^ School of New Energy, Nanjing University of Science and Technology, Jiangyin, China.; ^3^ Department of Mechanical and Energy Engineering, Southern University of Science and Technology, Shenzhen, China.; ^4^ State Key Laboratory of Organic-Inorganic Composites, Beijing University of Chemical Technology, Beijing, China.

## Abstract

Potassium (K) metal anodes suffer from uncontrolled solid electrolyte interphase evolution and isolated K accumulation, greatly hindering the construction of practical anode-free batteries. To date, systematic investigations on K stripping behavior and isolated K formation, despite being fundamentally important, are still lacking. Here, we develop an axially coordinated single-atom iron (Fe) anchored on hollow carbon bowls to synergize promoted K desorption and stress-adaptive ion transport. Serving as current collector modification, the FeN_4_O_2_ moiety optimizes K adsorption/desorption strength, regulates FSI^−^ decomposition, and suppresses electronically isolated K. Meanwhile, the mechanically compliant carbon-bowl scaffold mitigates volumetric strain during cycling, preserving interfacial integrity and accelerating desorption at the stripping frontier. Multimodal evidence from cryo–transmission electron microscopy, x-ray photoelectron spectroscopy depth profile, and theoretical calculations collectively reveals a bidirectional regulation to enhance both deposition uniformity and stripping reversibility. The anode-free K metal full cell delivers nearly 100 milliampere-hours per gram over 200 cycles at 200 milliamperes per gram, readily rivaling the state-of-the-art counterparts.

## Teaser

Axially coordinated single-atom Fe interface helps suppress isolated K, rendering reversible anode-free K metal batteries.

## INTRODUCTION

A potassium (K) metal anode, with a theoretical capacity of 685 mA·hour g^−1^ and a redox potential of −2.93 V versus standard hydrogen electrode, has emerged as an appealing candidate for next-generation high-energy batteries (*1*–*3*). During electrochemical cycles, metallic K undergoes repetitive plating/stripping, where the collective reversibility dictates the coulombic efficiency and cyclic stability of the electrode (*4*, *5*). In comparison with Li^+^, the lower Lewis acidity and larger ionic radius of K^+^ result in weaker solvation and faster ion mobility in common organic electrolytes. Nevertheless, the intrinsic reactivity of K metal and the tendency to form unstable solid-electrolyte interphases (SEIs) enable the electrochemically inactive “isolated K” (*6*–*8*). This would continuously consume the active K and/or electrolyte, thereby causing irreversible capacity fading. Recent studies offer compelling evidence that the metal stripping process predominantly governs the reversibility of alkali-metal electrodes, even in dendrite-free deposition states (*4*, *9*–*13*). In this sense, together with guaranteeing uniform deposition, ensuring complete stripping is the key to achieving durable K metal anodes.

The plating/stripping behavior of the K metal anode is strongly correlated with the electronic structure and interfacial chemistry of the current collector (*14*, *15*). Particularly in an anode-free configuration, where the cycling inventory of active K is supplied entirely by the cathode, interfacial reversibility over the current collector becomes even more crucial: Any loss of K during a single stripping event would directly reduce the total cell capacity at the device level (*16*). From the perspective of current collector design, copper (Cu)–, aluminum (Al)–, and carbon-based materials have been explored. It is noted that Al-based current collectors have certain merits in the compatibility with both anodes/cathodes, lightweight, low-cost, and mature manufacture (*17*). Typical current collectors suffer from poor electrolyte wettability and uneven SEI generation. Interfacial engineering has thus been identified as a promising route to improve reversibility. The state-of-the-art investigations readily demonstrated that the construction of electronic structure–tunable species, such as three-dimensional (3D) scaffolds, alloying layers, and single-atomic interfaces, could effectively regulate charge distribution and SEI composition (*18*, *19*). For instance, introducing Ni_3_S_2_/Ni_3_P heterostructures onto the current collector would elevate work function toward stabilized sodium (Na) cycling (*20*). The use of Co-N_4_ single-atom moieties was shown to enhance lithiophilicity and provide abundant nucleation sites by tailoring local electronic environments, thereby promoting more uniform lithium (Li) deposition (*21*, *22*). Our recent research endeavor revealed that metal atom-cluster cooperative sites would optimize K nucleation, enabling homogenized deposition by rationally modulating adsorption energetics at the interface (*23*). Nevertheless, these advances have predominantly focused on the deposition regulation, whereas the stripping behavior of the K metal, including dead K formation and SEI-mediated K^+^ transport, remains poorly understood despite emerging evidence from in situ microscopy that stripping was equally governed by interfacial chemistry and electronic continuity (*24*). During the plating/stripping of K, too strong K-interface interactions would render the trapping within the interface or SEI as electronically isolated K, while too weak interactions fail to sustain homogeneous plating (*25*, *26*). In this sense, Fe harvests a d-band center close to the Fermi level in the 3d transition-metal series, enabling effective electronic coupling with K without excessive binding strength. Such an intrinsic balance happens to satisfy the Sabatier criterion of moderate adsorption/desorption energetics (*27*), motivating us to extend the role of Fe single atoms from facilitating K deposition to governing stripping reversibility.

**Fig. 1.**
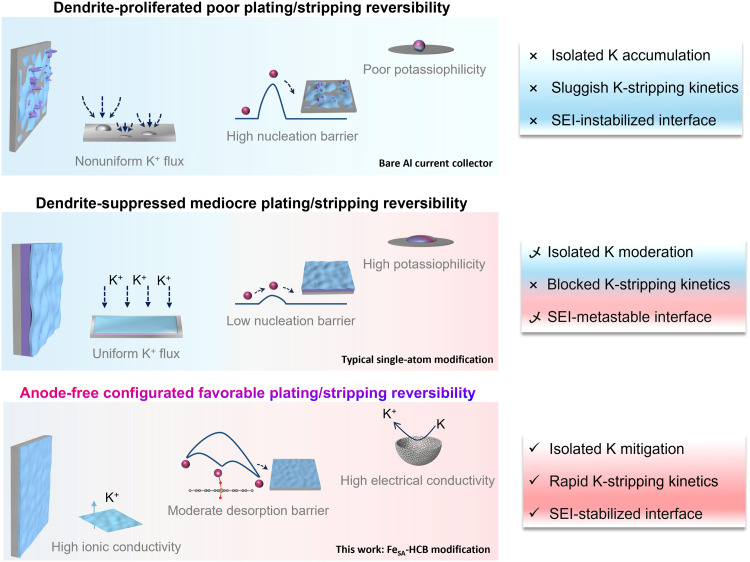
Schematic diagram showing the comparison of isolated K status and plating/stripping reversibility between bare Al and single-atom modified Al current collectors for anode-free batteries.

The K stripping process normally encompasses three fundamental stages: (i) interfacial oxidation (K → K^+^) requiring a conductive framework to allow charge transfer, (ii) K^+^ detachment affected by the local environment, and (iii) K^+^ transport across the SEI governed by SEI composition (*28*–*32*). While these steps are recognized, their interactive coupling to ensure effective K stripping has yet to be elucidated. Inspired by these considerations, we devise herein a unique FeN_4_O_2_ single-atomic moiety anchored on a hollow carbon-bowl framework (Fe_SA_-HCB) for Al current collector modification (Fig. 1). The open architecture of the carbon bowl ensures efficient electrical conductivity and uniform ion flux, while the axially oxygen (O)–coordinated Fe site enables smooth K migration with reduced desorption barriers and balanced adsorption/desorption features. Theoretical simulations also reveal more favorable FSI^−^ decomposition over the FeN_4_O_2_ moiety than that on the native Al_2_O_3_ surface, which facilitates the formation of inorganic-rich SEI. With the aid of exhaustive characterizations integrating cryo–transmission electron microscopy (cryo-TEM) visualization, biphenyl coloration assay, and x-ray photoelectron spectroscopy (XPS) depth profile, the tendency of dead K formation could be comparatively assessed. This evidence collectively provides a convergent mechanistic picture where the Fe_SA_-HCB interface readily promotes uniform K plating and crucially facilitates efficient K stripping. The thus-constructed anode-free full cell (PTCDA||Fe_SA_-HCB, where PTCDA refers to perylene-3,4,9,10-tetracarboxylic dianhydride) delivers stable cycling over 200 cycles at 200 mA g^−1^ with a reversible capacity of ~100 mA·hour g^−1^, competing favorably with the state-of-the-art counterparts. Our study represents the investigation of stripping-governed reversibility in anode-free K metal batteries and demonstrates that axially coordinated Fe single-atom interfaces could mitigate dead K formation.

## RESULTS

### Synthesis and characterization of Fe_SA_-HCB

**Fig. 2.**
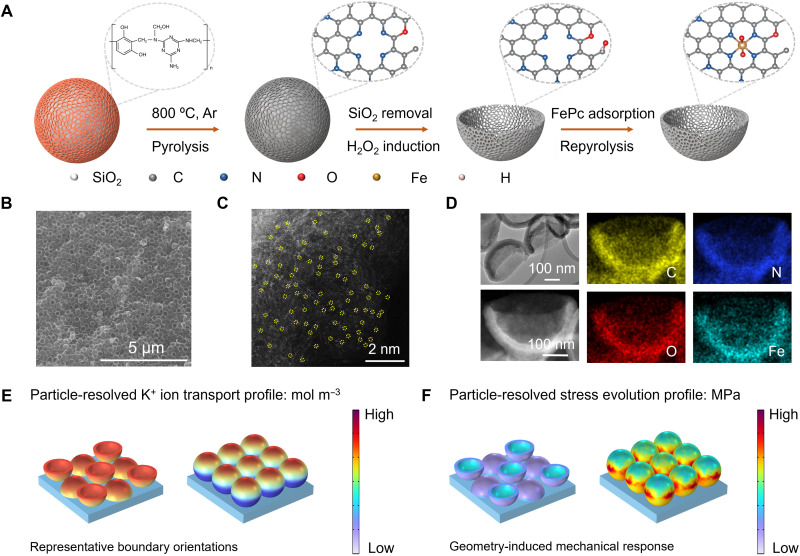
Synthesis and characterization. (**A**) Schematic illustration of the synthetic process of Fe_SA_-HCB. (**B**) Representative SEM image of HCB-H_2_O_2_. (**C**) Representative AC-HAADF-STEM image of Fe_SA_-HCB. (**D**) HAADF-STEM image and related EDS mapping of Fe_SA_-HCB. (**E**) Particle-resolved K^+^ concentration distribution profiles for HCB and hollow carbon sphere electrodes under representative boundary orientations (opening up and opening down). (**F**) Particle-resolved stress evolution profiles for HCB and hollow carbon sphere electrodes during K deposition, highlighting geometry-induced mechanical responses.

Figure 2A schematically depicts the synthetic procedures of Fe_SA_-HCB. Briefly, SiO_2_@melamine-resorcinol-formaldehyde polymer nanospheres were prepared by a modified Stöber route (the reaction system maintained in a weakly alkaline environment at pH 8 to 9), subsequently carbonized at 800°C under argon (Ar), and detemplated in 3 M NaOH (sodium hydroxide) to yield the HCB material (fig. S1) (*33*, *34*). The bowl-like versus sphere-like morphology is governed by the relative ratio of (resorcinol + formaldehyde) to tetraethoxysilane during synthesis (*35*). A tailored hydrogen peroxide (H_2_O_2_) hydrothermal treatment was applied to introduce additional porosity and oxygen functionalities (*36*). In this sense, reactive oxygen species would selectively oxidize edge and defect carbons while also attacking electronically enriched carbon sites activated by conjugation, promoting the formation of ─OH, C═O, and related O-containing groups. Subsequently, Fe was incorporated via ligand-assisted pyrolysis using iron phthalocyanine (FePc) as the Fe source. It is noted that the repyrolysis process enables Fe species to migrate into the N (nitrogen)/O–rich defective environment, where they could be stabilized via in-plane N coordination and axial O coordination originating from the H_2_O_2_-generated surface functionalities. A final acid washing would remove any residual Fe nanoclusters, yielding Fe_SA_-HCB affording atomically dispersed Fe moieties.

Scanning electron microscopy (SEM) and transmission electron microscopy (TEM) inspections reveal that our H_2_O_2_-treated samples (HCB-H_2_O_2_) display a homogeneous “bowl” morphology with an average outer diameter of ~300 nm and a shell thickness of ~30 nm (Fig. 2B and figs. S2 to S5), rendering a continuous mesoporous network to favor electrolyte wetting and ion transport. Simple statistical analysis of the shape population confirms a high fraction of complete bowl structure among the product, suggestive of synthetic controllability (fig. S6). The Fe incorporation upon the repyrolysis step would give rise to the formation of metallic nanoclusters, where subsequent acid washing helps remove these species, as witnessed by the x-ray diffraction (XRD) signals (fig. S7). Systematic investigations of H_2_O_2_ dosage and precursor ratio identify an optimum processing window (with the H_2_O_2_ dosage at 1.5 ml; precursor ratio of 1:1) to produce atomic Fe incorporation. Under such conditions, the Fe content reaches ~3.5 wt % in Fe_SA_-HCB (fig. S8 and tables S1 to S3). A representative aberration-corrected high-angle annular dark-field scanning transmission electron microscopy (AC-HAADF-STEM) image of Fe_SA_-HCB exhibits bright and isolated atomic contrasts, which could be attributed to the presence of highly dispersed Fe single atoms (Fig. 2C). Related energy-dispersive x-ray spectroscopy (EDS) mapping in Fig. 2D displays homogeneous spatial distributions of C, N, O, and Fe elements.

Although the primary pore size distribution of Fe_SA_-HCB remains centered at ~3.9 nm, comparable to that of HCB-H_2_O_2_ and bare HCB (fig. S9), the overall specific surface area decreases from 184.0 m^2^ g^−1^ (HCB-H_2_O_2_) to 172.2 m^2^ g^−1^ after Fe incorporation. This reduction is therefore not associated with pore collapse or size contraction. Still, it is more attributed to a decrease in accessible surface area arising from the anchoring of Fe single atoms on defect-rich pore walls. To rationalize the structural advantage of HCB, finite element method simulation based on COMSOL Multiphysics models was carried out for semiopen bowl and hollow-closed sphere geometries (fig. S10). The simulations were intentionally constructed as particle-resolved, few-body models, aiming to elucidate intrinsic geometry-induced ion transport and stress evolution within individual host architectures while decoupling these effects from electrode-scale stochastic packing. In the simulation framework, a K^+^ concentration gradient was imposed across the electrolyte domain, mimicking the realistic electrochemical scenario in which K^+^ migrates from the separator side toward the current collector–supported anode during plating, thereby giving rise to concentration polarization at the electrode/electrolyte interface. As for the simulated K^+^ concentration field, the bowl architecture generates a higher near-surface ion concentration gradient and better radial distribution over time, indicative of enhanced mass transport kinetics compared to the hollow sphere counterpart (Fig. 2E and fig. S11). Notably, the semiopen geometry effectively guides K^+^ flux from the separator-facing region toward the inner surface of the host, facilitating continuous ionic supply at the metal growth front adjacent to the current collector. Here, two opposite bowl orientations (opening up and opening down) were deliberately selected as representative boundary configurations to bracket the transport behavior expected from random orientations in practical composite electrodes. Moreover, force field simulations during K deposition manifest that the bowl geometry sustains substantially lower mechanical stress amid the modeling time frame (from 0 to 51 s), which could be ascribed to the more homogeneous ion influx and reduced local deposition-induced stress concentration under alleviated concentration polarization, suggesting improved accommodation of volume change and reduced tendency for SEI fracture (Fig. 2F and fig. S12). These results collectively imply the advances of the HCB for the Fe single-atom host.

**Fig. 3.**
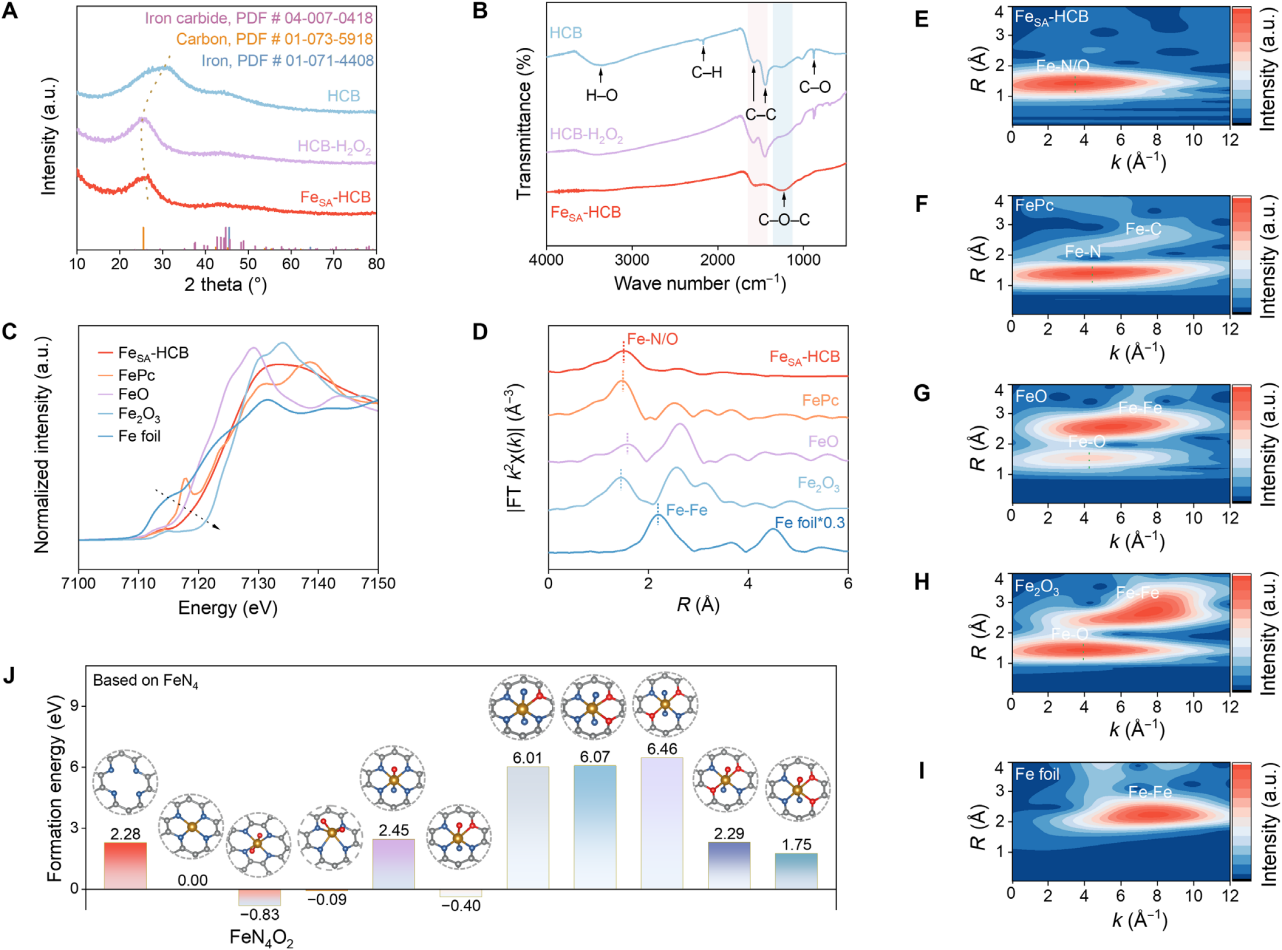
Electronic structure and local coordination environment analysis of the synthesized Fe_SA_-HCB. (**A**) XRD patterns of HCB, HCB-H_2_O_2_, and Fe_SA_-HCB. a.u., arbitrary units. (**B**) Fourier-transform infrared spectra of HCB, HCB-H_2_O_2_, and Fe_SA_-HCB. (**C**) Fe K-edge x-ray absorption near-edge structure profile of Fe_SA_-HCB. (**D**) Fe K-edge EXAFS fitting curves of Fe_SA_-HCB in the R space. FT, Fourier transform. Wavelet transform for the *k*^2^-weighted EXAFS spectra of (**E**) Fe_SA_-HCB, (**F**) FePc, (**G**) FeO, (**H**) Fe_2_O_3_, and (**I**) Fe foil. (**J**) Formation energy of different single-atom configurations derived by the DFT method.

Figure 3A shows the XRD patterns of bare HCB, HCB-H_2_O_2_, and Fe_SA_-HCB samples. The shifted (002) peak toward lower angles of HCB-H_2_O_2_ suggests the expansion of interlayer spacing relative to bare HCB. Raman spectra indicate noticeable defect densities in the carbon frameworks for all prepared materials (fig. S13), which are generally beneficial to enhancing electronic conductivity and interfacial charge transport by introducing additional charge delocalization pathways and active sites. Fourier-transform infrared spectra in Fig. 3B indicate a reduction of O─H signals with a preservation of C─O/C─O─C features, consistent with the surface oxidation step followed by partial dehydration via pyrolysis. XPS analysis helps elucidate the chemical states of Fe_SA_-HCB (figs. S14 and S15 and table S4) (*37*–*39*). The survey spectrum confirms the coexistence of C, N, O, and trace Fe species. In terms of the Fe 2p profile, no metallic Fe signal could be detected. Instead, characteristic Fe^2+^/Fe^3+^ signals (located at ~709.2/711.4 eV and 721.4/725.5 eV) dominate, indicating that Fe exists in oxidized, coordinatively bound states. Moreover, the deconvolution of the N 1s spectrum gives rise to pyridinic-N, pyrrolic-N/Fe─N, graphitic-N, and oxidized-N signals. In the high-resolution N 1s spectra, the Fe─N coordination signal partially overlaps with pyrrolic-N because of their close binding energy positions (typically ~399.5 to 400.5 eV). However, the presence of Fe─N coordination is corroborated by the concomitant Fe 2p signal and the absence of metallic Fe features. Meanwhile, the C 1s profile reveals a well-defined C─C, C─N, and O-containing carbon environment. Such a stable C/N/O framework provides abundant coordination sites and contributes to the stability of Fe centers.

Synchrotron-based x-ray absorption spectroscopy was further used to examine the coordination environment of thus-obtained Fe_SA_-HCB. As shown in the Fe K-edge x-ray absorption near-edge structure profile (Fig. 3C), the absorption edge of Fe_SA_-HCB is positioned in between those of Fe foil and Fe_3_O_4_, where quantitative fitting yields an average oxidation state of +2.7 (fig. S16). Notably, the measurements were conducted under evacuated and sealed conditions before data acquisition, minimizing contributions from adventitious surface–adsorbed species. Extended x-ray absorption fine structure (EXAFS) analysis discloses a dominant Fe─N scattering path and Fe─O path at ~1.49 Å, with well-reproduced *k*-space oscillations between experimental and fitted spectra, indicating reliable local structural assignment. Notably, no Fe─Fe scattering could be detected, verifying the absence of crystalline Fe clusters (Fig. 3D and figs. S17 and S18). The corresponding wavelet-transformed contour plots in Fig. 3 (E to I) further distinguish the local coordination signature of Fe_SA_-HCB from FePc, FeO, Fe_2_O_3_, or Fe foil. EXAFS fitting indicates that the average first-shell coordination number of Fe reaches ~5.9 (table S5), accompanied by low and stable Debye-Waller factors (σ^2^) and fitting residuals close to ideal values, suggesting that the coordinating N/O atoms are structurally stable. To gain insight into the coordination environments of single-atomic Fe, theoretical calculations based on density functional theory (DFT) were carried out to assess the formation energy of possible configurations. As displayed in Fig. 3J and fig. S19, the FeN_4_O_2_ configuration has the lowest formation energy in the examined computational space. Together, FeN_4_O_2_ with double axial oxygen coordination could be identified as the predominant motif in Fe_SA_-HCB.

### Identification and suppression of isolated K

Upon realizing the controllable synthesis of Fe_SA_-HCB, we examined how this interface would affect the dead K formation and K stripping reversibility. To evaluate the interfacial functionality of Fe_SA_-HCB in the K metal electrode, the prepared material was formulated into a coating slurry and casted onto commercial Al foil. The resulting modified current collector was used as the working electrode in half-cell and full-cell tests, enabling a direct correlation between atomic-scale coordination chemistry and current collector–level plating/stripping behavior. Given that K electrodes typically develop more reactive SEI than that of Li congeners, direct application of quantitative toolbox such as titration gas chromatography is challenging. In this sense, we deployed a multimodal protocol: cryo-TEM inspection to identify the SEI phase, electrochemical stripping measurement to probe the fraction of reactivatable K, and biphenyl coloration test to macroscopically detect exposed K. Note that the coloration originates from an electron transfer reaction in which metallic K reduces biphenyl to its radical anion state, producing a featured dark blue color

$$K+C_{12}H_{10}\to K^{+}+C_{12}H_{10}^{–}$$ (1)

Such a test provides a sensitive chemical probe to reveal electronically isolated K species that remained after cycling.

**Fig. 4.**
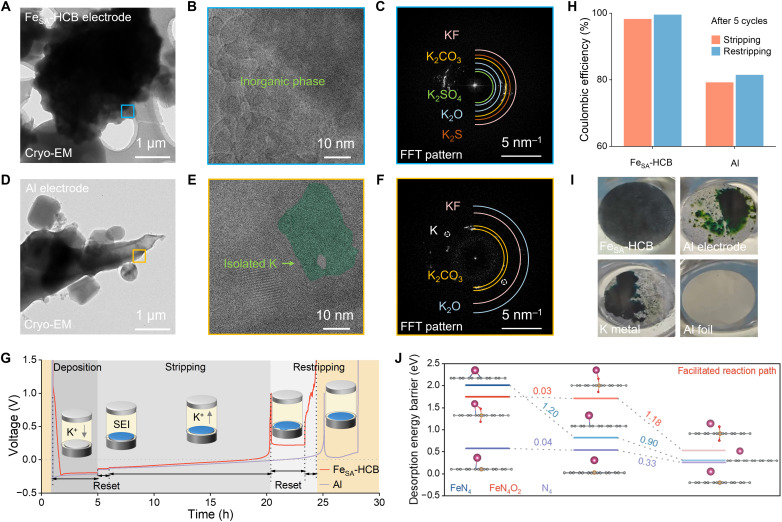
Suppression of isolated K. (**A** to **C**) Cryo-TEM images of Fe_SA_-HCB electrode, showing low- and high-magnification views with corresponding FFT (fast Fourier transform) pattern. (**D** to **F**) Cryo-TEM images of Al electrode, showing low- and high-magnification views with corresponding FFT pattern. (**G**) Two consecutive stripping curves of Fe_SA_-HCB and Al electrodes at 0.5 mA cm^−2^. h, hours. (**H**) Comparison of coulombic efficiency of first stripping versus restripping after five cycles for Fe_SA_-HCB and Al. (**I**) Digital photos showing chromogenic behaviors of different supports (Fe_SA_-HCB electrode, Al electrode, K metal, and Al foil). (**J**) Desorption energy barriers of K ions on FeN_4_, FeN_4_O_2_, and N_4_ substrates.

Cryo-TEM observation of K electrodes that experienced 20 cycles at 0.5 mA cm^−2^ reveals distinct SEI structures. As shown in Fig. 4 (A to C) and fig. S20, the Fe_SA_-HCB–derived K electrode harvests a continuous inorganic-rich SEI comprising KF, K_2_O, K_2_S, K_2_CO_3_, and K_2_SO_4_, affording no detectable isolated K crystallites. In contrast, the Al-derived electrode displays dendritic protrusion and isolated K accumulation, accompanied by KF, K_2_O, K_2_CO_3_, and substantial amounts of organic components (Fig. 4, D to F). Mechanically fragile K_2_CO_3_/KHCO_3_ domains form around isolated K and undergo tearing under cycle-induced stress (fig. S21), exposing organic lamellae and forming an unstable SEI. To quantify the reversibility, we carried out a two-step stripping test: depositing 2 mA·hour cm^−2^ K at 0.5 mA cm^−2^ and conducting the first stripping and, after certain rest, performing the second stripping. As shown in Fig. 4G, Fe_SA_-HCB enables shorter initial stripping times. Previous studies have shown that in current collector–based metal deposition systems, the working electrode potential was not strictly defined by a reversible K/K^+^ redox couple, and thus, plating/stripping potentials might deviate from 0 V owing to interfacial polarization and SEI resistance (*16*, *40*, *41*). Upon five cycles, it delivers the first/second stripping coulombic efficiencies of 98.2%/99.5% (Fig. 4H and fig. S22), whereas the Al-based electrode merely achieves 79.2%/81.4%. These attained data not only indicate favorable K stripping efficiency via Fe_SA_-HCB but also reveal certain reactivation of isolated K by rest, in agreement with a recent report (*42*).

Figure 4I shows results of the biphenyl coloration assay. The control test readily confirmed that typical inorganic SEI species (KF, KOH, and K_2_CO_3_) did not cause coloration (fig. S23). Before the biphenyl coloration test, the collected electrodes were thoroughly rinsed with dimethoxyethane solvent to remove residual electrolyte salts. Metallic K enables an instant color change to deep blue. As displayed by the digital photos, the Al-based electrode manifests pronounced blue/green patches after 20 cycles, implying the presence of residual isolated K. On the contrary, the Fe_SA_-HCB–derived electrode induces no coloration to the naked eye, demonstrating suppressed isolated K formation. The consistent trends obtained from these tests establish that axial O-coordinated Fe sites substantially promote reversible K stripping and thus mitigate the dead K. To elucidate the essence of efficient stripping behavior, DFT computations (*43*–*45*) of the K desorption energy barrier were carried out. As depicted in Fig. 4J and fig. S24, K normally migrates from electron-rich N sites to less electronegative C sites for desorption, but the direct N → C step affords a high-energy barrier. Introducing an axial O allows the creation of a low-energy intermediate (N → O: 0.03 eV); such a pathway would markedly enhance the desorption probability. In comparison with conventional FeN_4_, FeN_4_O_2_ also achieves a balanced adsorption-desorption profile.

### SEI composition and interfacial chemistry

To rationalize the intrinsic origin of superior stripping reversibility and dead-K suppression ability of Fe_SA_-HCB, we systematically examined the plating/stripping interface and the resulting SEI composition. First, wettability measurements using the 4 M potassium bis(fluorosulfony)imide (KFSI)–based electrolyte reveal notable differences among the substrates. Fe_SA_-HCB– and HCB-modified current collectors respectively exhibit low contact angles of 10° and 15°, whereas bare Al shows a larger angle of 48.4° (fig. S25). For the case of HCB-H_2_O_2_ without Fe incorporation, it shows a similarly reduced contact angle, indicating that the improved wettability mainly originates from oxygen-containing functional groups rather than Fe atomic sites. The good electrolyte wettability is in support of homogeneous ion transport during plating/stripping. Meanwhile, digital photos of electrodes after deposition of 5.0 mA·hour cm^−2^ K show that Fe_SA_-HCB enables the formation of dense and smooth K coverage. In contrast, discontinuous K accumulations with incomplete surface coverage could be observed on the bare Al surface (fig. S26). Upon 30 plating/stripping cycles at 0.2, 0.5, or 2.0 mA cm^−2^ (figs. S27 to S32), the Fe_SA_-HCB–based current collector retains highly uniform K deposition. Comparatively, Al shows pronounced cavities and protrusions even at 0.2 mA cm^−2^, together with severely uneven stripping interfaces, corroborating its incompetence to guide stable SEI evolution.

**Fig. 5.**
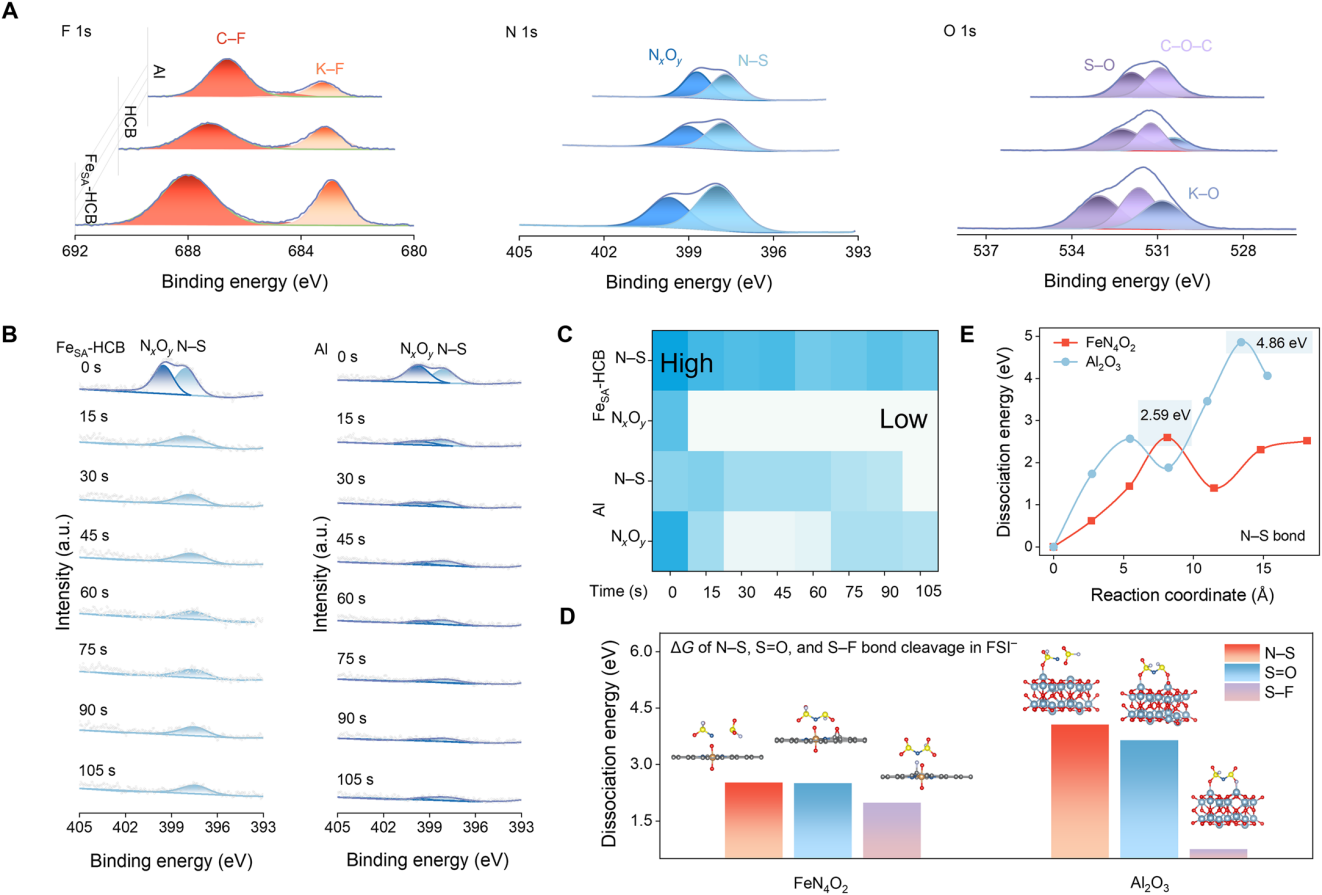
Composition analysis and formation mechanism of the interfacial SEI. (**A**) High-resolution XPS spectra of F 1s, N 1s, and O 1s for Fe_SA_-HCB, HCB, and Al. (**B**) XPS depth profiling and (**C**) quantitative analysis of the N 1s spectra for Fe_SA_-HCB and Al. (**D**) Comparison of DFT-calculated dissociation energy barriers of FSI^−^ on Fe_SA_-HCB and Al substrates. (**E**) Reaction coordinate of N─S bond dissociation.

To uncover the chemical basis underlying these morphological disparities, XPS depth profiling was performed. As evidenced by the surface XPS spectra in Fig. 5A, Fe_SA_-HCB hosts substantially higher fractions of inorganic K─F, K─O, and N─S species, while Al retains abundant C─F and N*_x_*O*_y_* organic residues. The depth profiles dissect the vertical continuity of SEI components. As shown in Fig. 5 (B and C), the organic N*_x_*O*_y_* signal disappears rapidly on Fe_SA_-HCB with sputtering, leaving only the N─S peak. In the meantime, a stable KF peak at 682.7 eV remains even beyond 45-s sputter (fig. S33), suggesting the formation of continuous inorganic-rich SEI. In comparison, Al maintains N*_x_*O*_y_* signals throughout the sputter depth, indicative of a chemically disordered SEI. These depth-resolved results are consistent with the foregoing analysis on stripping reversibility. It is worth noting that despite a substantial amount of K deposition during cycles, the SEI chemistry is not solely determined by the instantaneous K/electrolyte interface. Instead, it reflects the history-dependent interphase evolution established during early nucleation and subsequent plating/stripping, where the underlying substrate could regulate electrolyte decomposition pathways and therefore imprint distinct SEI chemistries (*46*, *47*).

To gain insight into the SEI compositional differences, theoretical computations of FSI^−^ adsorption and dissociation on FeN_4_O_2_ or Al_2_O_3_ surfaces were performed (Fig. 5D and figs. S34 and S35). Note that the Al_2_O_3_ surface was selected because the native oxide layers on the commercial Al current collector could dominate its interfacial chemistry. As reflected by the adsorption energy calculation, Al_2_O_3_ binds FSI^−^ more strongly (−2.26 eV) as compared to FeN_4_O_2_ (−1.00 eV). A further comparison of Gibbs free energy with respect to the cleavage of FSI^−^ internal bonding (N─S, S═O, and S─F) between Al_2_O_3_ and FeN_4_O_2_ reveals substrate-dependent selectivity: On FeN_4_O_2_, the N─S and S═O cleavage barriers are relatively low, favoring pathways to yield KF, K_2_O, and sulfur oxide inorganic species. To further validate this conclusion, climbing-image nudged elastic band calculations (Fig. 5E) were performed for the N─S bond breakage, explicitly capturing the transition states and confirming the reduced kinetic barriers on FeN_4_O_2_ relative to Al_2_O_3_ (*48*). On Al_2_O_3_, the S─F cleavage is comparatively facile. This is driven by the strong Al─F interactions that effectively “pin” the F atoms. However, the overall overadsorption of FSI^−^ on Al_2_O_3_ hinders certain dissociation steps, producing less amounts of inorganic residuals. Together, FeN_4_O_2_ manifests a moderate adsorption strength and low cleavage barrier for FSI^−^ to enable tailored generation of mechanically robust inorganic SEI. Such substrate-regulated interfacial reactions are consistent with recent reports demonstrating that metal nucleation behavior and SEI evolution can be jointly governed by substrate chemistry and electrolyte decomposition, which ultimately determine the stability and ion-transport properties of the interphase (*49*, *50*).

### Probing the electrochemical kinetics

**Fig. 6.**
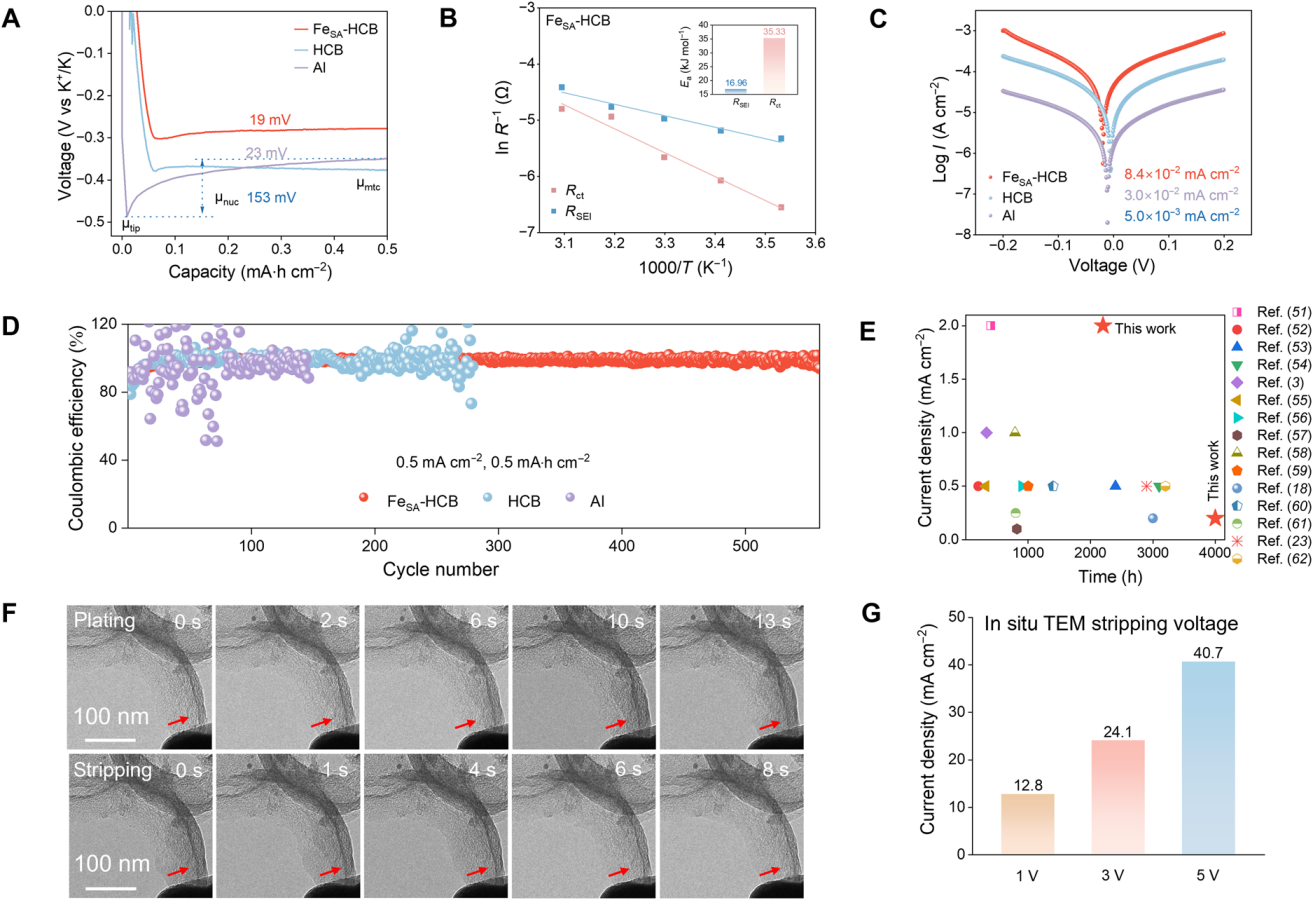
Electrochemical kinetics analysis. (**A**) Voltage profiles of K nucleation at 0.5 mA cm^−2^. (**B**) Activation energy comparison of *R*_ct_ and *R*_SEI_ for Fe_SA_-HCB electrodes. (**C**) Tafel plots. (**D**) Coulombic efficiency plots as a function of cycle number for K||current collector half cells at 0.5 mA cm^−2^. (**E**) Comparison of the cycle life of symmetric cells with the Fe_SA_-HCB–modified Al current collector and those of previously reported K metal anodes. (**F**) In situ TEM observation of a typical K plating/stripping process for Fe_SA_-HCB. (**G**) Current density at different stripping voltages (1, 3, and 5 V).

After establishing that Fe_SA_-HCB could effectively suppress inactive K accumulation and promote robust inorganic-rich SEI formation, we next evaluated its electrochemical kinetics under operating conditions to clarify how its interfacial advantages translate into plating/stripping reversibility. At identical current densities, the Fe_SA_-HCB–modified current collector consistently delivers the lowest nucleation overpotential across the entire range of 0.2 to 5.0 mA cm^−2^, registering only 5 to 136 mV as compared with 12 to 153 mV for HCB (Fig. 6A and fig. S36). Bare Al fails to form a stable nucleation plateau at ≥1.0 mA cm^−2^, underscoring its intrinsically sluggish interfacial kinetics. These differences arise not only from current redistribution afforded by the open bowl geometry but also from the electronic/chemical effects of the FeN_4_O_2_ axial coordination center, which lowers the interfacial energy barrier and offers accessible sites for synchronized K nucleation, lateral migration, and growth. Electrochemical impedance spectroscopy (EIS) data collected under different temperatures also indicate that Fe_SA_-HCB exhibits the lowest activation energy (figs. S37 and S38). To identify the kinetically crucial step, we further extracted the activation energies associated with SEI resistance (*R*_SEI_) and charge-transfer resistance (*R*_ct_) in Fe_SA_-HCB symmetric cells (Fig. 6B). The *R*_SEI_ displays a small activation energy (16.96 kJ mol^−1^), whereas *R*_ct_ reaches 35.33 kJ mol^−1^, indicating that charge transfer, not the SEI composition, is the rate-determining step. Notably, even after substantial K deposition, the effective interface is not an ideal K/electrolyte boundary but a dynamically evolving K/SEI/electrolyte module, rendering charge transfer across this interface the key kinetic step. This trend is quantitatively consistent with Tafel analysis, linear sweep voltammetry, and cyclic voltammetry data (Fig. 6C and figs. S39 and S40), showing markedly higher exchange current density, lower polarization, and more stable peak shapes for Fe_SA_-HCB in contrast to those of bare Al. The higher exchange current density for Fe_SA_-HCB therefore confirms its intrinsically faster interfacial kinetics beyond any geometric surface area contribution. Galvanostatic intermittent titration technique measurements further corroborate this by displaying a higher apparent K^+^ diffusion coefficient for Fe_SA_-HCB (fig. S41). These results confirm that single-atomic Fe moieties help accelerate charge transfer pathways to enable rapid and reversible K deposition. Quantitative analysis of the average voltage hysteresis in symmetric cells again reveals notably lower interfacial polarization for Fe_SA_-HCB in comparison with bare Al during the prolonged cycling (table S6).

The question whether this kinetic advantage persists over extended cycling is explained through in situ EIS and distribution of relaxation time (DRT) analysis. Deconvolution of time constants reveals that Fe_SA_-HCB maintains smoothly evolved *R*_ct_ and *R*_SEI_ values for 10 to 50 cycles, whereas Al exhibits continuously increasing and strongly fluctuating impedances (figs. S42 and S43), indicative of repeated SEI rupture and reconstruction. The persistence of kinetic differences after prolonged cycling arises from the heterogeneous nature of K stripping: Residual K and preformed SEI continuously modulate the interfacial environment, preventing the interface from becoming a pure K/electrolyte boundary. The hollow carbon-bowl architecture of Fe_SA_-HCB provides spatial confinement and mechanical buffering that maintain a stable SEI and homogeneous ion flux throughout cycling, whereas planar Al suffers from progressive SEI degradation. The impedance evolution correlates directly with the cycling behavior (Fig. 6, D and E, and figs. S44 and S45): Fe_SA_-HCB enables ~580 cycles in half cells at 0.5 mA cm^−2^ and >4000-hour operation in symmetric cells at 0.2 mA cm^−2^. It also enables outstanding durability even under stringent current/capacity conditions (2.0 mA cm^−2^/2.0 mA·hour cm^−2^), which sustains ~300 cycles (half cell) or >2100 hours (symmetric cell) (figs. S46 and S47) (*3*, *18*, *23*, *51*–*62*). To evaluate the interface response under K^+^ flux variation, we managed to cycle Fe_SA_-HCB symmetric cells at a fixed areal capacity (1 mA·hour cm^−2^) while progressively increasing the current density from 0.5 to 10 mA cm^−2^ (fig. S48). Moreover, stable cycling was also achieved under 100% depth-of-discharge conditions, underscoring the robustness of the Fe_SA_-HCB interface. To further verify morphological preservations after prolonged cycling, Fe_SA_-HCB collected from cells cycled at 0.5 mA cm^−2^ was examined by HAADF-STEM (figs. S49 and S50). The hollow carbon-bowl architecture remains intact with only residual K deposits, with multiangle AC-HAADF-STEM imaging clearly showing isolated Fe single-atomic sites. These results demonstrate that Fe_SA_-HCB forms a mechanically stable, electrically contiguous, and ionically conductive interface that preserves the kinetic benefits over long-term operation.

To visualize the structural evolution at the atomic level, we carried out in situ TEM study. Because carbon bowls have hollow, thin-walled shells with multiple open edges whose spatial orientation relative to the tungsten probe is difficult to precisely control, achieving reliable “probe-carbon shell-K metal” electrical contact is highly challenging. Occasional local shell discontinuities thus arise from unavoidable beam-geometry constraints rather than intrinsic structural instability (fig. S51). Once stable electrical contact is established, K rapidly enters the carbon shell within 0 to 13 s and uniformly wets the inner surface. Subsequent deplating occurs within ~8 s, during which the carbon bowl returns to its original shape with a minimal volume change (Fig. 6, F and G). These observations confirm that the carbon bowl acts as an effective mechanical buffer to prevent SEI fracture during stress fluctuations and render reversible plating/stripping, in good agreement with our finite element method simulations.

### Full-cell performance evaluation

Guided by foregoing insights, we lastly evaluated the performance of the Fe_SA_-HCB–modified current collector in K metal batteries. All electrochemical evaluations were conducted under comparable laboratory-scale cathode loadings to ensure that performance differences primarily reflect interfacial mechanisms rather than loading-induced artifacts (*63*). At the device level, the typical full cell was constructed using a KFSI-based electrolyte and a PTCDA cathode. Compared with typical KPF_6_ or KTFSI salts, KFSI features a more favorable solvation structure and higher anion reactivity, enabling the formation of a low-impedance, inorganic-rich SEI over K metal (*30*). PTCDA, as a representative organic carbonyl cathode, offers a well-defined redox mechanism, good structural stability, and moderate operating potential, making it a widely adopted cathode material for K metal batteries (*31*, *32*). The full cell was first assembled using a PTCDA cathode and composite anode preloaded with 5 mA·hour cm^−2^ K metal (PTCDA||K@Fe_SA_-HCB, PTCDA||K@HCB, and PTCDA||K@Al). Across 0.1 to 2.0 A g^−1^, PTCDA||K@Fe_SA_-HCB exhibits the best rate performance among the tested systems, retaining a capacity of ~150 mA·hour g^−1^ at 2.0 A g^−1^ (figs. S52 and S53). This performance arises from the combination of fast interfacial charge transfer and stable SEI chemistry, allowing homogeneous K plating/stripping even at elevated current densities. To correlate capacity retention with interfacial evolution, the early-cycle impedance behaviors of PTCDA||K@Fe_SA_-HCB and PTCDA||K@Al were comparatively studied (figs. S54 and S55). Fe_SA_-HCB shows a progressive impedance decrease during charging, reflecting the transformation of SEI into a thinner, more conductive inorganic-dominated structure as K^+^ migrates from the cathode to the anode. During discharge, the impedance remains stable, demonstrating unobstructed electron and ion transport. In contrast, Al exhibits continuously increasing SEI resistance and erratic *R*_ct_/diffusion components, corroborating a brittle SEI structure, excessive electrolyte decomposition, and the formation of a thick, ion-insulating SEI.

**Fig. 7.**
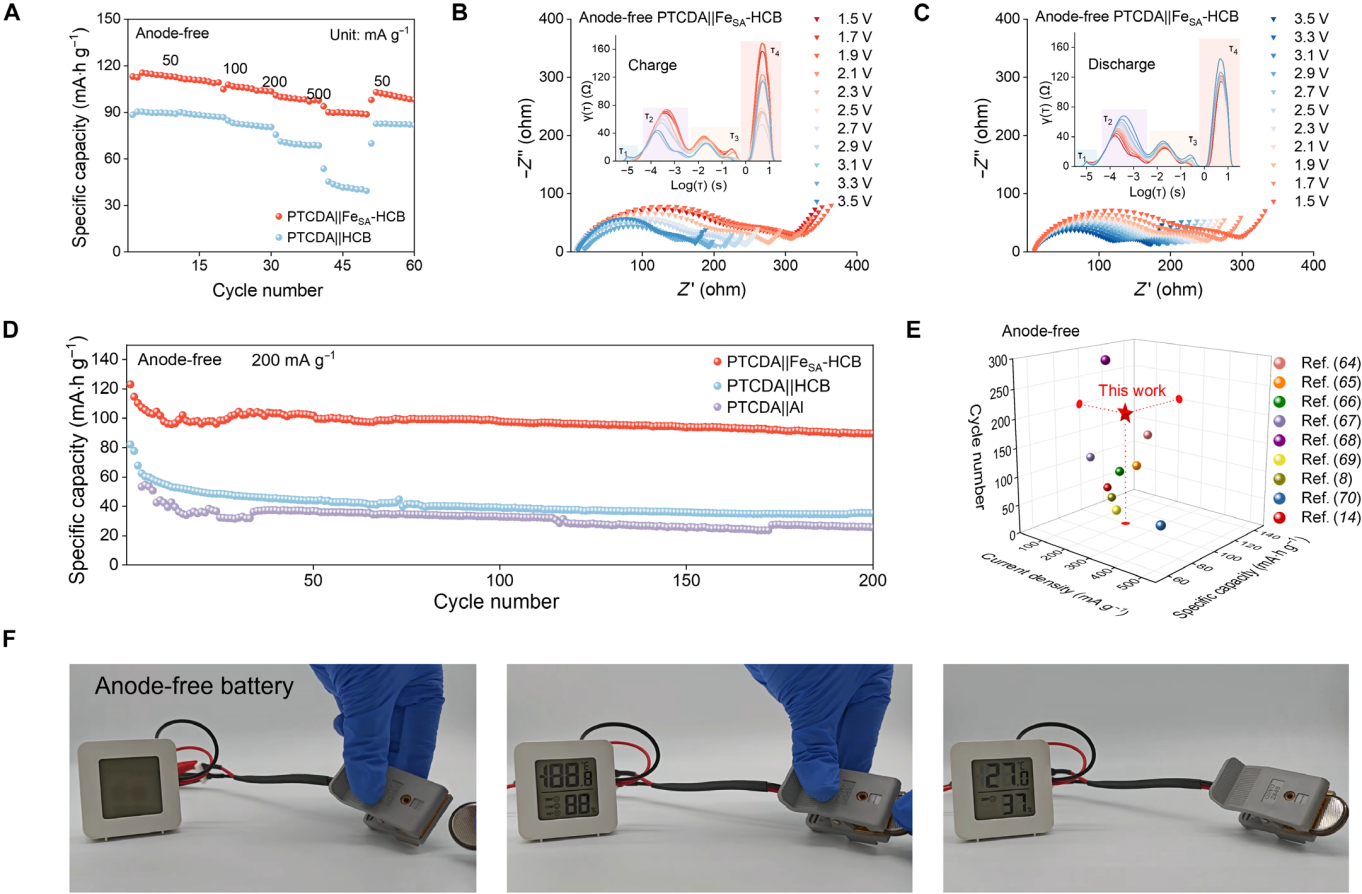
Electrochemical performances of K metal full cells. (**A**) Rate performances. (**B** and **C**) In situ EIS profiles of the anode-free PTCDA||Fe_SA_-HCB charge/discharge cycle, with the inset displaying the corresponding DRT analysis results. (**D**) Cycling performances at 200 mA g^−1^. (**E**) Comparison of the cycle number of anode-free full cells with the Fe_SA_-HCB–modified Al current collector and those of previous reports. (**F**) Digital photos showing a coin cell–based anode-free K metal battery powering a miniaturized temperature/humidity indicator.

Subsequently, we evaluated the performance of full cells affording the anode-free configuration (N/P = 0), where the cathode serves as the sole K source and no metallic K is initially present at the anode side. In comparison with K-metal full cells with excess K reservoirs, the anode-free configuration imposes a far more stringent requirement on stripping reversibility, as any electronically isolated K would directly translate into irreversible capacity loss. The construction of anode-free configuration further underscores the advantages of Fe_SA_-HCB in governing interfacial stability and stripping reversibility. Upon prepotassiation of PTCDA and SEI activation of Fe_SA_-HCB, the resulting anode-free PTCDA||Fe_SA_-HCB full cell delivers well-defined and fully reversible redox peaks (fig. S56), indicating that the interface remains active even under zero excess K conditions. Our Fe_SA_-HCB also affords good rate capability in anode-free conditions (Fig. 7A), further confirming that fast ion/electron transport can be maintained even without a K reservoir. Impedance and DRT analyses reveal the underlying origin of this behavior: During charging, the Fe_SA_-HCB interface exhibits a gradual and monotonic decrease in total impedance (Fig. 7, B and C, and fig. S57). During discharge, the impedance displays temporally coherent features in all four DRT time-constant regions, demonstrating stable SEI integrity, unobstructed electron transport, and minimal dead K accumulation even as the available K inventory becomes limited. In contrast, the Al-based anode-free cell undergoes a sharp impedance collapse during charging and maintains high resistance values during discharging. Benefiting from this well-regulated interfacial evolution, PTCDA||Fe_SA_-HCB harvests a capacity of ~100 mA·hour g^−1^ for 200 cycles at 200 mA g^−1^ (Fig. 7, D and E), outperforming the rapid degradation seen with Al and readily rivaling the state-of-the-art anode-free K and Na systems reported to date (*8*, *14*, *64*–*70*). In response, a benchmarking comparison is summarized in table S7. This robust electrochemical reversibility translates into practical operability that the assembled anode-free PTCDA||Fe_SA_-HCB device can successfully power a miniaturized temperature/humidity indicator (Fig. 7F).

## DISCUSSION

In summary, we have put forward a structure-directed interfacial engineering strategy to simultaneously enable uniform K deposition, reversible K stripping, and stable SEI evolution. By integrating axially coordinated single-atom Fe sites with a hollow carbon-bowl scaffold capable of stress accommodation, the engineered current collector interface modulates K adsorption/desorption energy and catalytic decomposition pathways of FSI^−^. Such a regulation helps suppress isolated K accumulation, accelerate stripping kinetics, and maintain a robust inorganic-rich SEI. Synergistic evidence from theoretical simulations, cryo-TEM inspection, XPS depth profile, in situ impedance mapping, and anode-free cell validation establishes a direct mechanistic link between the atomic coordination environment, interfacial chemical evolution, and macroscopic cycling reversibility. Collectively, these findings offer a generalizable pathway toward high-efficiency anode-free K metal batteries and provide a meaningful paradigm for broader alkali-metal energy storage devices.

## MATERIALS AND METHODS

### Synthesis of Fe_SA_-HCB

Hollow carbon bowls (HCBs) were synthesized via a modified Stöber-assisted polymerization-carbonization route. Briefly, an ammonia-ethanol-water mixture (9 ml of NH_3_·H_2_O, 32 wt %; 210 ml of ethanol; 30 ml of deionized water) was stirred for 30 min, followed by the dropwise addition of 8.1 ml of tetraethyl orthosilicate over 30 min to form silica spheres. Resorcinol (1.2 g) and formaldehyde (1.68 ml) were then added and stirred for 30 min, after which melamine (1.2 g) and additional formaldehyde (0.84 ml) were sequentially introduced to construct a melamine-resorcinol-formaldehyde polymer shell. After stirring for 24 hours, the SiO_2_@MRFPS precursor was collected, washed, and dried at 80°C for 12 hours, followed by carbonization at 800°C for 4 hours under Ar (5°C min^−1^). The silica core was removed by etching in 3 M NaOH for 36 hours to obtain HCBs. To construct axially coordinated single-atom Fe sites, HCBs were first oxidatively etched in a mixed solution of H_2_O_2_ (1.5 ml, 30%), ethanol (25 ml), and deionized water (25 ml) via hydrothermal treatment at 100°C for 5 hours to introduce oxygen-containing defect sites. Subsequently, 100 mg of treated HCB and 100 mg of FePc were dispersed in 40 ml of ethanol, sonicated for 2 hours, and stirred for 24 hours to allow molecular-level adsorption. The FePc@HCB precursor was pyrolyzed at 800°C for 2 hours under Ar (5°C min^−1^), followed by acid leaching in 1 M HCl to remove residual Fe clusters, yielding Fe_SA_-HCB with atomically dispersed Fe sites anchored on HCBs.

### Characterizations

The crystal phases were probed by XRD (Bruker A8 Advance Diffractometer) using Cu-Kα radiation (λ = 1.5406 Å). The morphological features of samples were inspected by SEM (Hitachi S-4800 field-emission SEM operated at an accelerating voltage of 10 kV). Raman spectra were recorded on a Jobin Yvon LabRAM HR800 instrument with an excitation wavelength of 632 nm. XPS measurements were performed using an Escalab 250Xi Spectrophotometer. The content of Fe was obtained by inductively coupled plasma analysis (OPTIMA 8000).

### Cryo-TEM sample preparation

Cryo-TEM characterization was performed using a FEI Talos-S equipped with a Gatan 698 cryo-transfer holder. The samples underwent 40-hour electrochemical cycling and then experienced disassembling within an Ar-filled glove box [<0.1 ppm (parts per million) of O_2_/H_2_O]. Materials were scraped off from the current collector surfaces and were dispersed onto TEM Cu grids under inert conditions. Grids were subsequently transferred into the cryo-TEM chamber using a cryogenic shuttle system. All imaging and analytical procedures were carried out at cryogenic temperatures. The elemental mapping was executed in STEM mode.

### In situ TEM sample preparation

In situ TEM measurements were performed using a transmission electron microscope (Thermo Fisher Scientific Talos, 120 kV) and a Nanofactory TEM sample holder (Zepyo Technology Co., Ltd., China). The yolk-shell Fe_SA_-HCB nanocomposite, which was attached to a gold mesh, served as the working electrode. Metallic K, placed on the tungsten tip, functioned as both the counter and reference electrodes. The thin potassium oxide (K_2_O) layer on the K surface acted as the solid electrolyte. Both the gold mesh and tungsten tip, containing metallic K, were mounted onto the TEM sample holder and protected under an Ar atmosphere before being inserted into the specimen chamber. Once the K_2_O-covered K electrode, controlled by a micro motor, made contact with the free end of the selected nanocomposite, a constant voltage ranging from −5 to 5 V was applied across the sample to facilitate the potassiation and depotassiation of the Fe_SA_-HCB nanocomposite.

### AC-TEM

Specifically, all TEM imaging and EDS mapping were conducted using a JEOL ARM-200F electron microscope equipped with a cold field emission gun, spherical aberration (Cs) corrector for the condenser lens, and active vibration and magnetic damping systems. The system offers STEM image resolutions of 0.078 nm at 200 kV, 0.111 nm at 80 kV, and 0.136 nm at 60 kV, with an energy resolution better than 0.5 eV at 200 kV. The microscope was operated at an accelerating voltage of 200 kV with a STEM probe current of 17 pA, a probe size of 0.133 nm, a convergence angle of 20.6 mrad, and a collection angle of 54 to 220 mrad. Advanced detection modes including annular dark-field, annular bright-field, and bright-field modes were used. The system was further equipped with a Gatan 1095 complementary metal-oxide semiconductor camera, a Gatan 1065 energy filter, and a high-efficiency energy-dispersive x-ray spectrometer (JED-2300T) with a large solid angle of 0.97 sr.

### Electrochemical measurements

The electrochemical performances were investigated by using CR2032 coin cells assembled in an Ar-filled glove box with oxygen and water contents less than 0.01 ppm. Asymmetric cells were assembled by separating the current collector and K metal with a piece of glass fiber (Whatman). Symmetric cells were prepared with two pieces of identical current collectors (Fe_SA_-HCB, HCB, and Al) that were predeposited with 5.0 mA·hour cm^−2^ K metal. A fixed amount (110 μl) of electrolyte and 4 M KFSI were added in dimethoxyethane without any additives. The asymmetric cells were subjected to three formation cycles between a voltage range of 0 to 1 V at a current density of 50 μA cm^−2^ for activation before testing. To evaluate the K plating/stripping behavior, the asymmetric cells were tested at different current densities and stripped to 1.0 V. Galvanostatic cycling measurements were carried out on the basis of symmetric cells under repeated charge/discharge for 1 hour, respectively. EIS profiles and Tafel curves were carried on a CHI660E electrochemistry workstation.

As for K metal full cells, PTCDA electrodes were fabricated by slurry-casting [mixing PTCDA, super P, and polyvinylidene difluoride (5%) binder at a weight ratio of 7:2:1] on bare Al foil. The mass loading of each PTCDA electrode was 1.1 to 1.5 mg cm^−1^. Before assembling full cells, PTCDA electrodes were prepotassiated in half cells at 0.01 mA g^−1^. The separators and electrolyte without any additives used in full cells were the same as those in half cells. Before assembling the anode-free full cells, the PTCDA cathode was prepotassiated at a current density of 0.5 A g^−1^, while the Fe_SA_-HCB–modified current collector was electrochemically activated within 0 to 1 V to establish a stable interfacial layer.

The activation energy (*E*_a_) could be calculated on the basis of the Arrhenius equation

$$k=A\text{exp}\left(-\frac{E_{a}}{\mathrm{RT}}\right)$$ (2)

where *k* is the rate constant, *R* represents the molar gas constant [8.314 (J/(mol·K)], and *T* indicates the thermodynamic temperature.

The equation can be simplified as follows

$$\frac{1}{R_{\text{SEI}}}=A\text{exp}\left(-\frac{E_{a}}{\mathrm{RT}}\right)$$ (3)

$$\frac{1}{R_{\text{ct}}}=A\text{exp}\left(-\frac{E_{a}}{\mathrm{RT}}\right)$$ (4)

where *R*_SEI_ and *R*_ct_ were derived from the equivalent circuit model.

### Computational details

DFT calculations were performed using the Vienna Ab initio Simulation Package (*53*). The Perdew-Burke-Ernzerhof functional with the generalized gradient approximation (*54*) was used to describe the exchange and correction effects between electrons for atoms. All models were built on the basis of a 5 × 3 × 1 graphene supercell with a 15-Å-thick vacuum. The DFT-D3 method (*3*) was adopted to compute the van der Waals correction for these two-dimensional materials. The plane-wave cutoff energy, energy convergence, and force threshold were set as 450 eV, 10^−5^ eV, and 0.01 eV Å^−1^, respectively. The Monkhorst-Pack *k*-point grid was set as 2 × 2 × 1 for the Brillouin zone. The formation energy was computed for various species with different coordination environments and structures, as follows

$$E_{\text{form}}=E_{\text{material}}-E_{\text{base}}-\sum_{i}n_{i}u_{i}$$ (5)

where *E*_material_, *E*_base_, *n_i_*, and *u_i_* represent the free energy of investigated material, the free energy of the base material (FeN_4_), the number of atoms of type *i*, and the corresponding chemical potential of type *i* element, respectively. Notably, *n_i_* > 0 and *n_i_* < 0 mean type *i* atoms added and removed, respectively. The stripping energy was computed according to the energy difference between material with K ion adsorbed and material as well as K atom. The charge density difference and Bader charge methods were implemented to reveal the charge transfer between material and K atom. For the adsorption and dissociation of FSI^−^ simulations, both the zero-point vibrational energy and enthalpic correction were considered to compute the adsorption energy and dissociation energy. The climbing-image nudged elastic band (*55*) calculations were conducted to disclose the dissociation process and barrier energy of the N─S bond in FSI^−^.

### Numerical simulation method

The numerical simulations presented in this work are designed to resolve the intrinsic chemomechanical responses of individual carbon hosts with distinct geometries, rather than to reconstruct the full composite electrode microstructure. In practical electrodes, carbon hosts are randomly oriented and embedded in heterogeneous matrices composed of conductive additives and polymeric binders, making deterministic nanoscale reconstruction experimentally inaccessible without extensive 3D tomography.

Accordingly, simplified particle-resolved models with a limited number of representative geometries are adopted to provide identifiable parameters and mechanistically interpretable trends. The opening-up and opening-down configurations of the carbon bowl are treated as bounding cases that bracket the range of ion transport and stress responses expected from randomly oriented particles. While multilayer or large-area electrode-scale models could be constructed, such complexity would not alter the geometry-dependent transport and stress characteristics resolved here but would substantially increase computational cost and obscure the underlying physical mechanisms.

The numerical simulation is implemented by COMSOL Multiphysics 6.3. The primary focus of the simulation is to model the differences in diffusion rates and expansion coefficients between carbon bowls and hollow carbon spheres. Key parameters include the following: K^+^ diffusion coefficient in carbon: 10^−14^ m^2^/s; K^+^ expansion rate in carbon: 20%; conductivity of 4 M KFSI/dimethoxyethane electrolyte: 5 mS/cm; dimensions: Both carbon bowls and hollow carbon spheres have an outer diameter of 300 nm and a wall thickness of 30 nm.

The simulation involves constructing 3D models of carbon bowls and hollow carbon spheres, arranged in a 3 × 3 regular array. In the concentration simulation, K^+^ ions flow at a uniform rate from the top surface of the carbon bowls and spheres, resulting in a nonuniform concentration distribution within the models. After obtaining the concentration distribution, the results are imported into the solid mechanics module to calculate the expansion stress distribution caused by K^+^ ion intercalation.

The concentration field model (ion diffusion) can be expressed as follows. On the basis of the steady-state continuity equations and the law of mass conservation, we have the control equation for ion diffusion

$$\frac{\text{∂}\left(\in C\right)}{\text{∂}t}+\nabla \cdot \left(-D_{\text{eff}}\nabla C+\mathrm{uC}\right)=R$$ (6)

where *u* is the flow velocity (which is zero in this case), *R* is the source/sink term (e.g., chemical reactions) [mol/(m^3^·s)], *C* is the species concentration, ϵ is the porosity (which is 1 in this case), and *D* is the diffusion coefficient.

The stress field model (mechanical equilibrium) can be expressed as follows. The mechanical deformation due to ion insertion/extraction is modeled using elastic theory with eigenstrain (chemically induced strain)

∇·σ+f=0 (7)

where the Cauchy stress tensor (σ) is given by

$$\sigma_{x}=\frac{E}{\left(1+v\right)\left(1-2v\right)}\left[\left(1-v\right)\epsilon_{x}+v\left(\epsilon_{y}+\epsilon_{z}\right)\right]$$ (8)

$$\sigma_{y}=\frac{E}{\left(1+v\right)\left(1-2v\right)}\left[\left(1-v\right)\epsilon_{y}+v\left(\epsilon_{x}+\epsilon_{z}\right)\right]$$ (9)

$$\sigma_{z}=\frac{E}{\left(1+v\right)\left(1-2v\right)}\left[\left(1-v\right)\epsilon_{z}+v\left(\epsilon_{x}+\epsilon_{y}\right)\right]$$ (10)

The relevant parameters are as follows: $\sigma_{x},\sigma_{y},\sigma_{z}$: normal stresses (Pa); $\in_{x},\in_{y},\in_{z}$: normal strains (dimensionless); *E*: Young’s modulus (Pa); *v*: Poisson’s ratio.

The strain components can be written as

$$\in =\frac{1}{2}\left[\nabla u+{\left(\nabla u\right)}^{T}\right]$$ (11)

$$\in^{C}=\frac{V}{3}\left(c-c_{0}\right)I$$ (12)

where $c_{0}$ is the initial reference concentration, *V* is the partial molar volume of the concentration, and *c* is the concentration value. This coupled concentration-stress model captures the ion diffusion under stress gradients and the stress evolution due to chemomechanical expansion.

### Distribution of relaxation time analysis

DRT analysis transforms EIS data from the frequency domain to the time domain, enabling precise extraction of relaxation time parameters for distinct electrochemical processes. The DRT methodology models electrochemical systems as a continuum of series-coupled polarization processes exhibiting distributed relaxation time constants, which is derived from the following formula

$$Z\left(\omega \right)=R_{\infty }+\int_{0}^{\infty }\frac{\gamma \left(\tau \right)}{1+j\omega \tau }d\tau$$ (13)

where τ is the specific relaxation time (time constant), *Z*(ω) represents the total impedance, *R*_∞_ is the basic ohm impedance, and γ(τ) is the relaxation distributional function.
