# Supplementary material for: Axially coordinated single-atom interface mitigates isolated K toward highly reversible anode-free K metal batteries
Source: Sci Adv. 2026 Jul 24;12(30):eaef1038. doi: 10.1126/sciadv.aef1038 (PMC13398483; doi:10.1126/sciadv.aef1038)
Supplement: Supplementary file 1 — Figs. S1 to S57 Tables S1 to S7 [file sciadv.aef1038_sm.pdf]

Science Advances
AAAS

Supplementary Materials for

Axially coordinated single-atom interface mitigates isolated K toward highly reversible anode-free K metal batteries

Qian Liu *et al.*

Corresponding author: Yongbiao Mu, muyb2021@mail.sustech.edu.cn; Le Yu, yule@mail.buct.edu.cn; Jingyu Sun, sunjy86@suda.edu.cn

Sci. Adv. **12**, eaef1038 (2026)
DOI: 10.1126/sciadv.aef1038

This PDF file includes:

Figs. S1 to S57
Tables S1 to S7

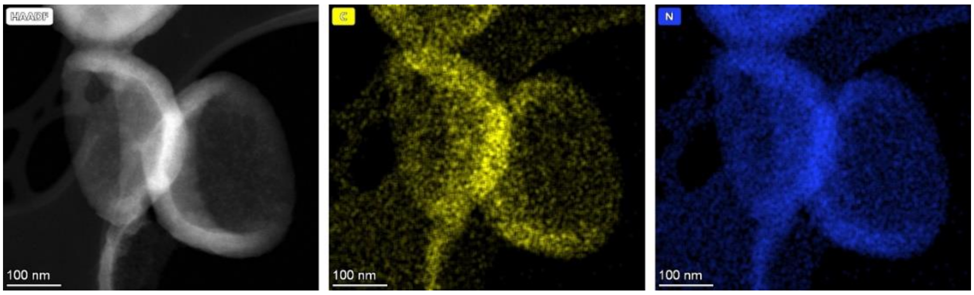

Fig. S1. HAADF-STEM image and corresponding element mappings of HCB.

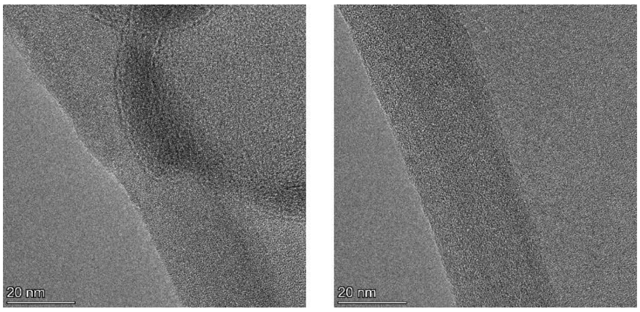

Fig. S2. High-resolution TEM images of HCB-H_2O_2 showing the shell thickness.

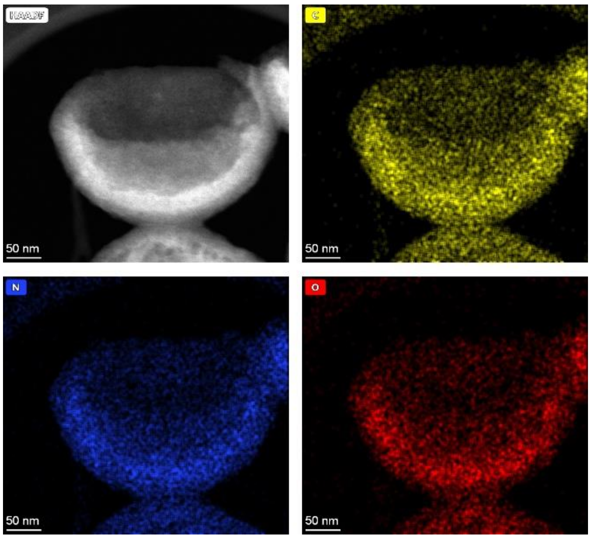

Fig. S3. HAADF-STEM image and corresponding element mappings of carbon hemispheres after H_2O_2 treatment.

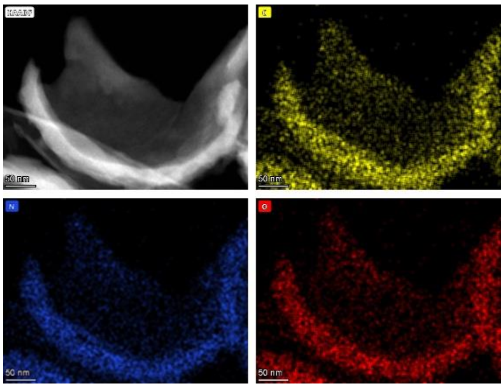

Fig. S4. HAADF-STEM image and corresponding element mappings of sub-hemispherical carbon fragments after H_2O_2 treatment.

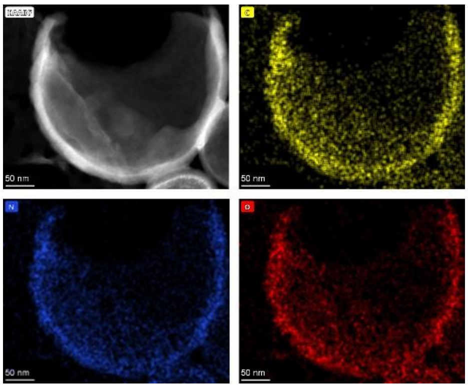

Fig. S5. HAADF-STEM image and corresponding element mappings of over-hemispherical carbon shells after H_2O_2 treatment.

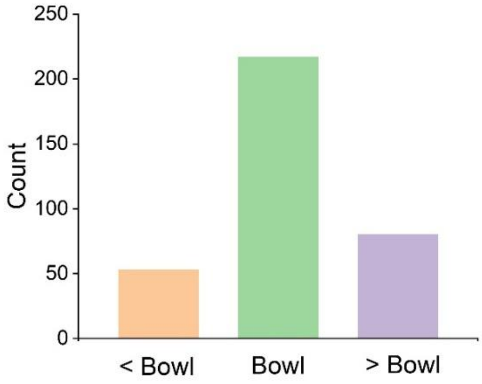

Fig. S6. Frequency distribution of sub-hemispherical carbon fragments, carbon hemispheres and over-hemispherical carbon shells.

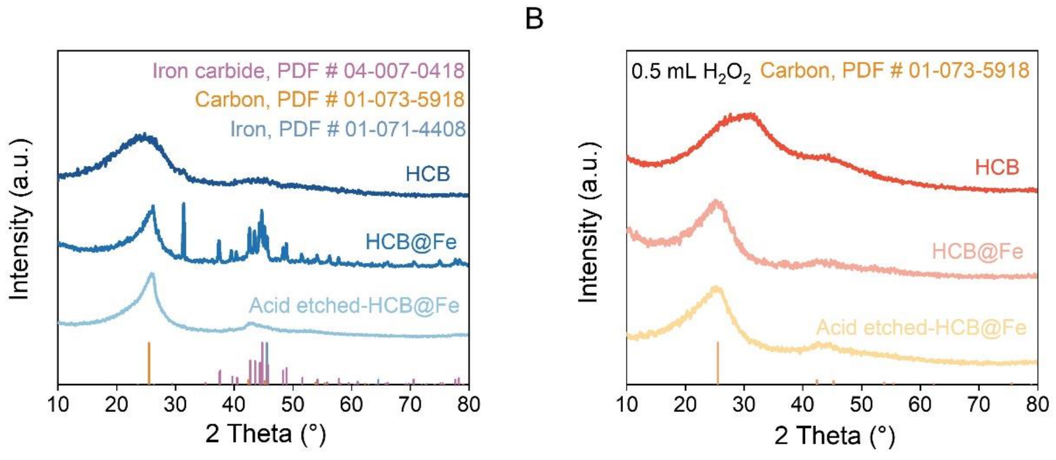

Fig. S7. XRD patterns of HCB, HCB@Fe, and acid etched-HCB@Fe, showing (A) conventionally synthesized and (B) after pore formation using H_2O_2.

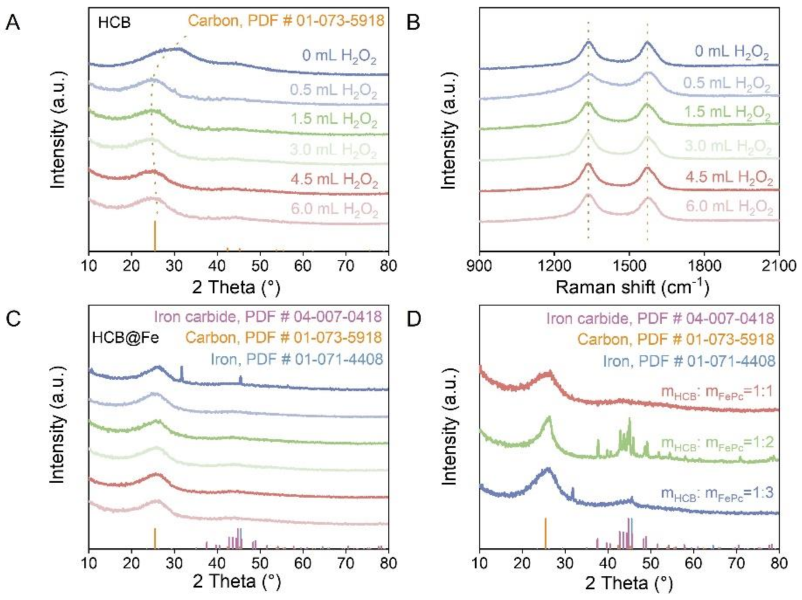

Fig. S8. (A) XRD patterns and (B) Raman spectra of HCB with tunable pore structures achieved by varying H_2O_2 concentrations, along with (C) XRD patterns after metal loading. (D) XRD patterns of Fe_{SA}-HCB synthesized by adjusting the concentration of metal precursors.

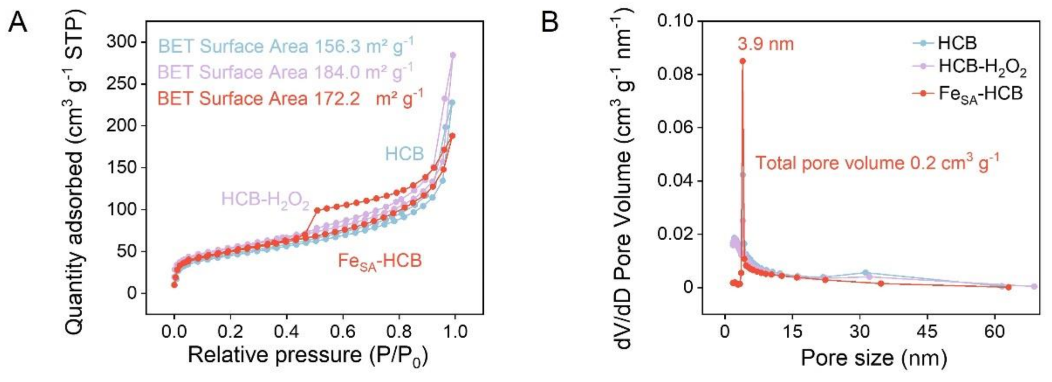

Fig. S9. (A) N_2 adsorption/desorption isotherms and (B) pore-size distribution curves of the HCB, HCB-H_2O_2 and Fe_{SA}-HCB.

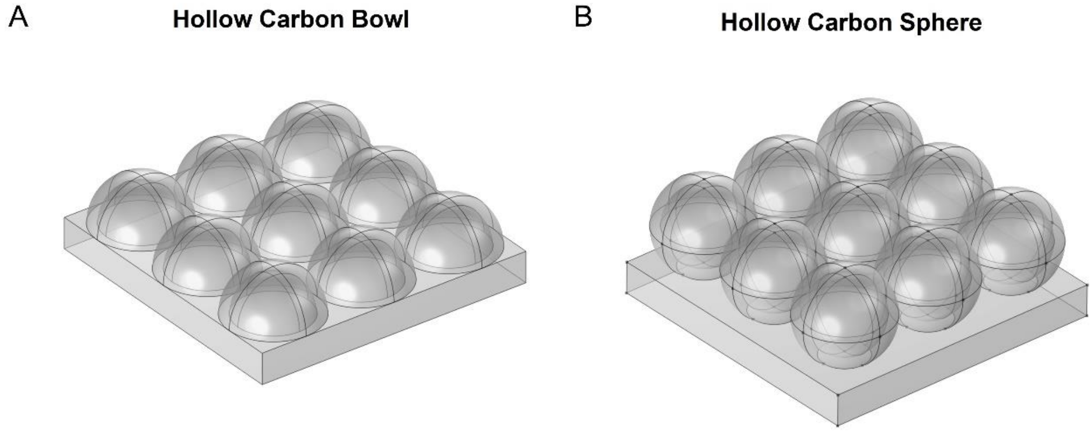

Fig. S10. The constructed COMSOL simulation models of (A) hollow carbon bowl (HCB) electrode and (B) hollow carbon sphere (HCS) electrode.

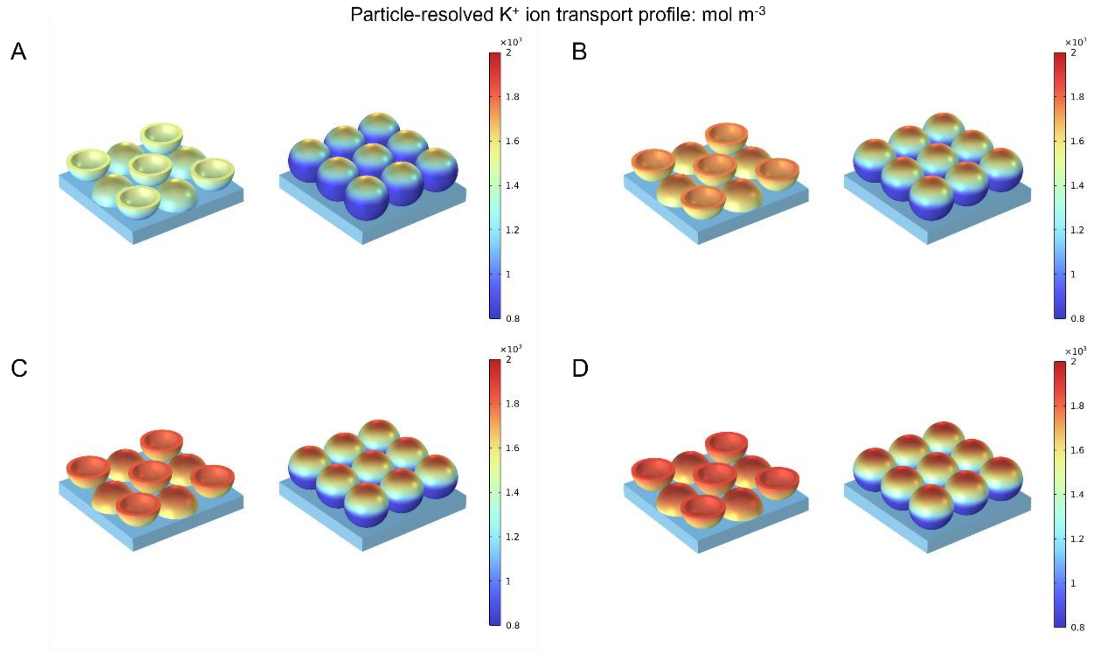

Fig. S11. The snapshots of K^+ concentration distribution profile.

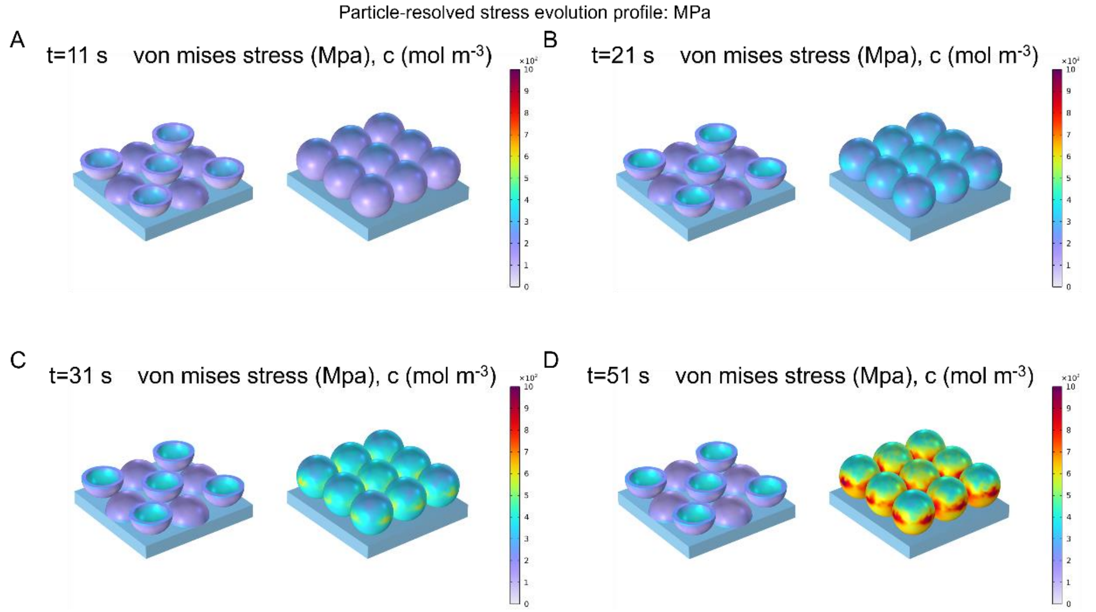

Fig. S12. The snapshots of stress profile on the K-deposited layer.

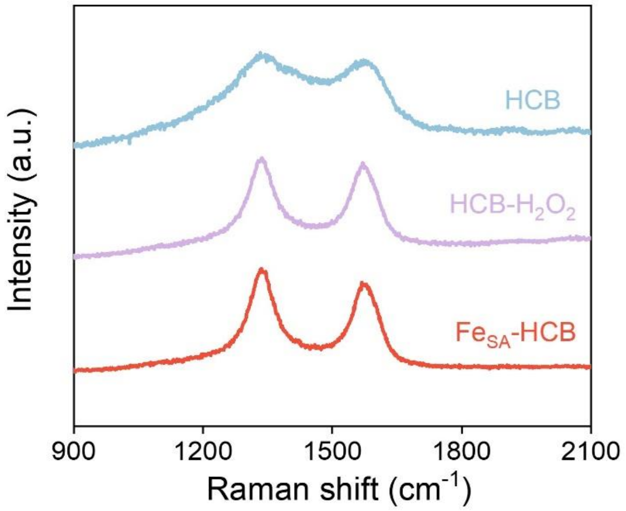

Fig. S13. Raman spectra of the prepared HCB, HCB-H_2O_2 and Fe_{SA}-HCB.

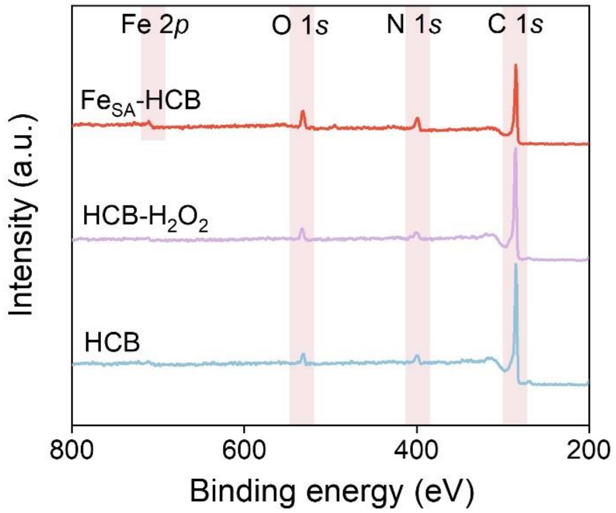

Fig. S14. XPS survey spectra of the prepared HCB, HCB-H_2O_2 and Fe_{SA}-HCB.

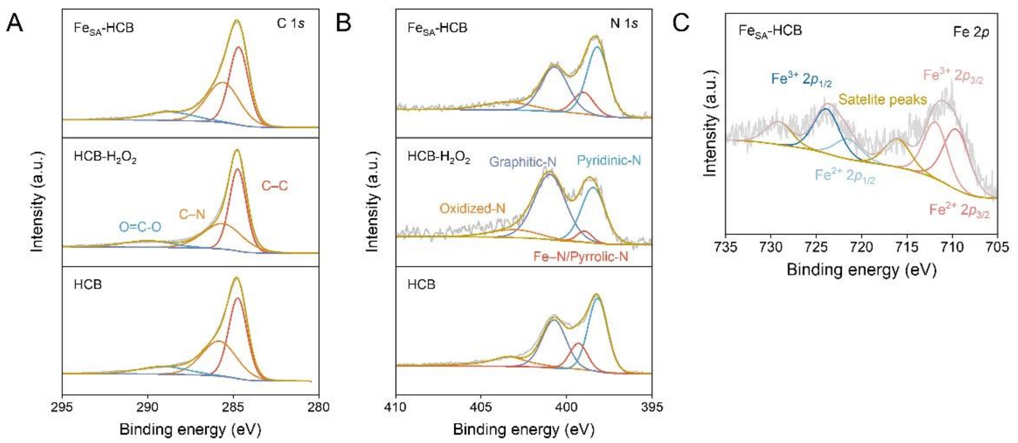

Fig. S15. The high-resolution XPS (A) C 1*s* spectra, (B) N 1*s* spectra and (C) Fe 2*p* spectra.

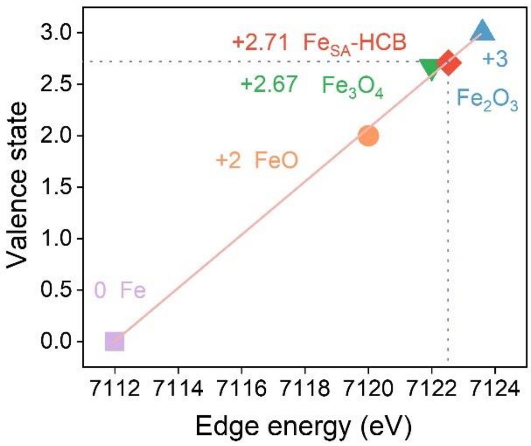

Fig. S16. Iron valence state of the Fe$_{SA}$-HCB sample.

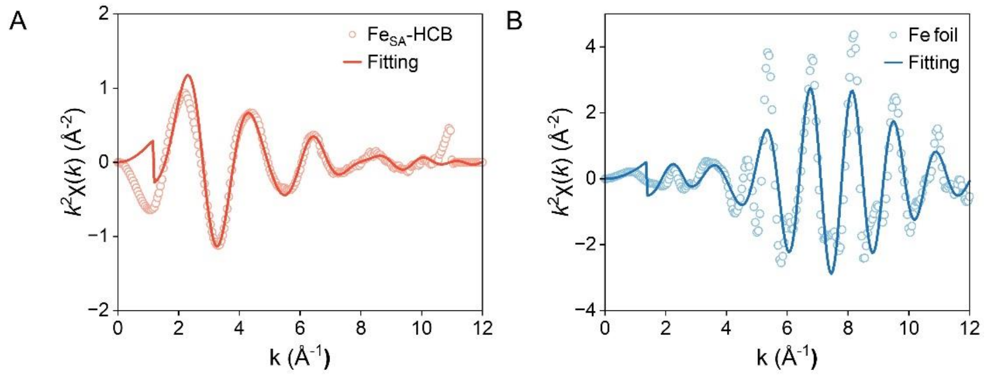

Fig. S17. The EXAFS profile at k space and corresponding fitting of (A) Fe_{SA}-HCB and (B) Fe foil.

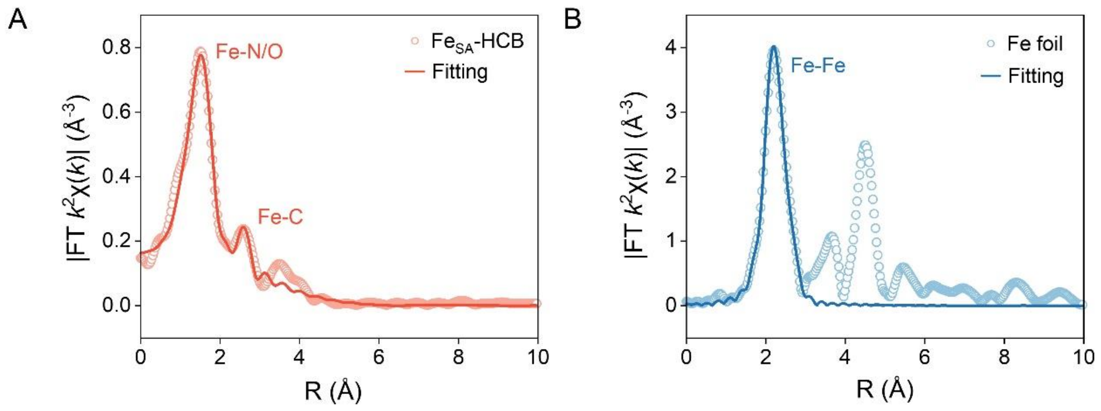

Fig. S18. The FT k^2-weighted EXAFS profile of (a) Fe_{SA}-HCB and (b) Fe foil.

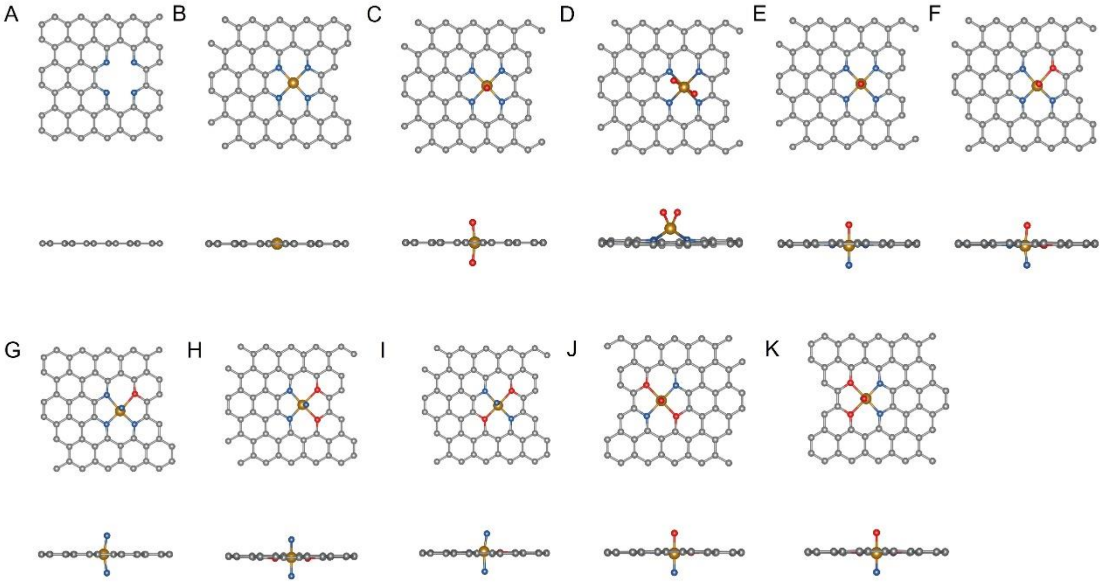

Fig. S19. Top and side views of the different configurations (A) N_4, (B) FeN_4, (C) FeN_4–O_2 (contralateral side O atoms), (D) FeN_4–O_2 (ipsilateral side O atoms), (E) FeN_4–N–O (contralateral side O and N atoms), (F) FeN_3O–N–O (contralateral side O and N atoms), (G) FeN_3O–N_2 (contralateral side N atoms), (H) FeN_2O_2–N_2 (contralateral side N atoms), (I) FeN_2O_2–N_2 (contralateral side O atoms in plane), (J) FeN_2O_2–N–O (contralateral side O atoms in plane), (K) FeN_2O_2–N–O (ipsilateral side N atoms in plane).

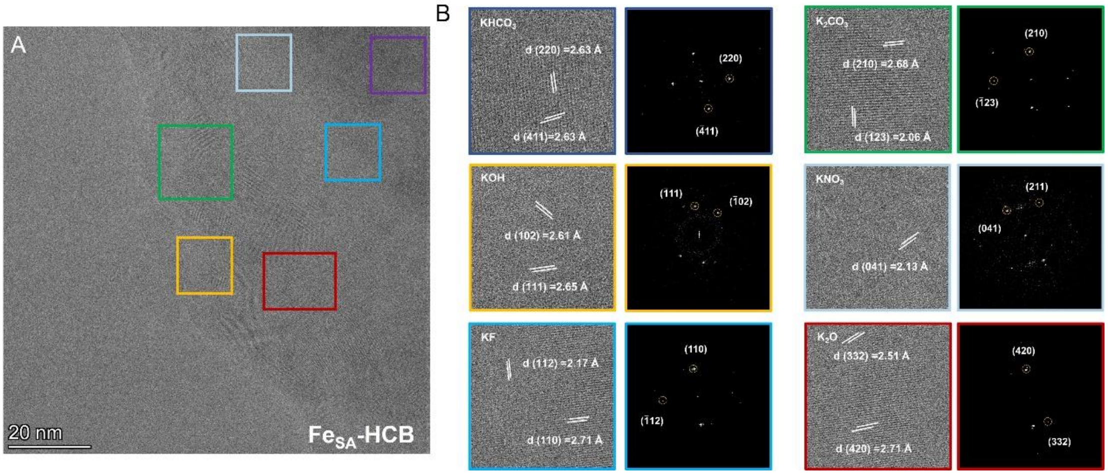

Fig. S20. Component analysis of Fe_{SA}-HCB derived SEI by cryo-TEM, showing representative HRTEM images of $KHCO_3$, K_2CO_3, KOH, KNO_3, KF and K_2O with their indexed FFT patterns.

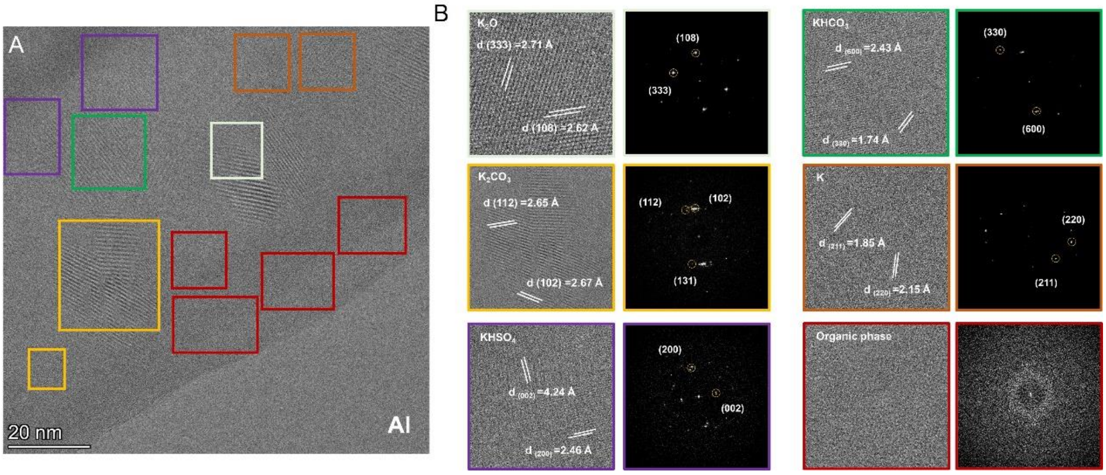

Fig. S21. Component analysis of Al derived SEI by cryo-TEM, showing representative HRTEM images of $KHCO_3$, K_2CO_3, $KHSO_4$, K and organic phases with their indexed FFT patterns.

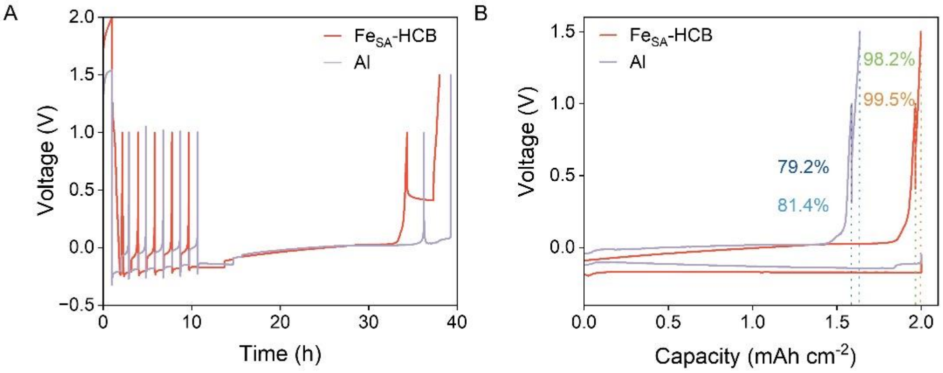

Fig. S22. (A) Time-voltage profile of the second stripping process after 5 cycles at 0.5 mA cm^{-2}. (B) Corresponding capacity−voltage profile.

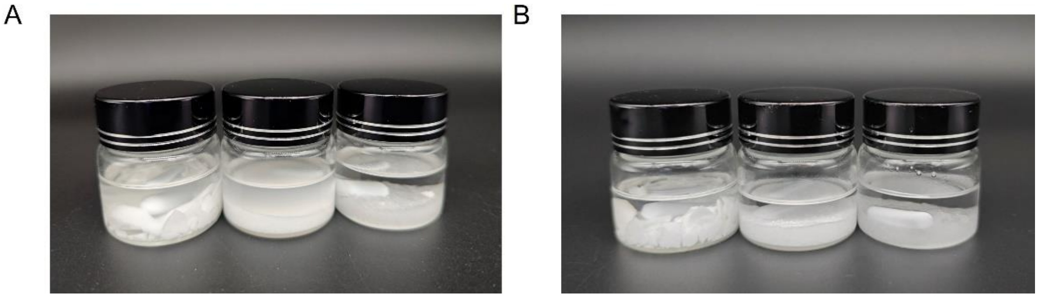

Fig. S23. Digital photos of KOH, K_2CO_3, and KF in biphenyl/DME solution (A) after stirring and (B) after standing.

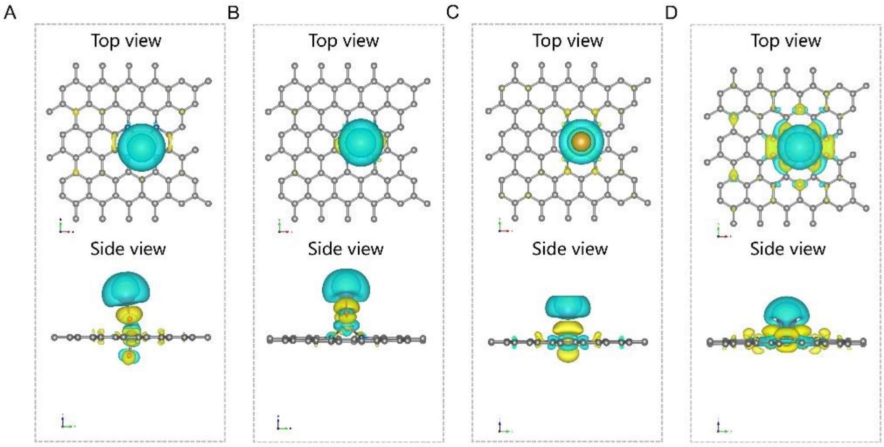

Fig. S24. Charge density differences between K and (A) FeN_4-O_2 (anti-side), (B) FeN_4-O_2 (syn-side), (C) FeN_4 and (D) NC. The yellow and cyan contour with the isosurface value of 0.001 e/bohr^3 represents the accumulated and reduced charge density, respectively.

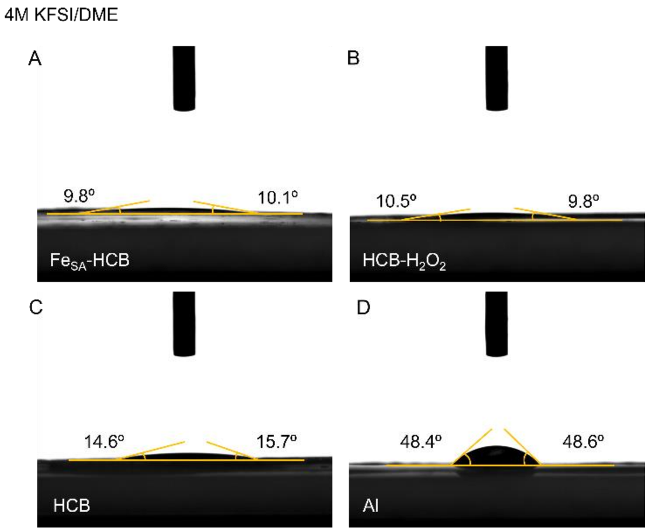

Fig. S25. Static contact angle measurements of the electrolyte on the different current collector surfaces.

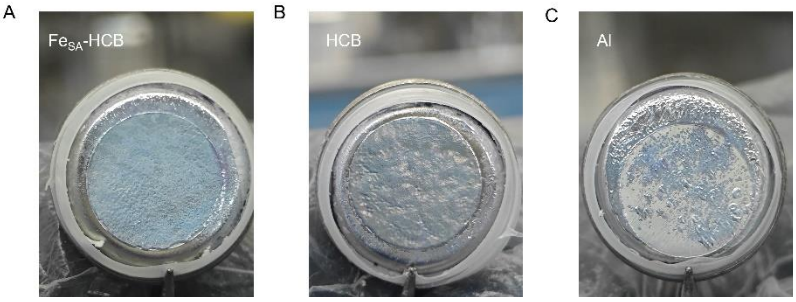

Fig. S26. Digital photos of different current collectors after deposition of 5.0 mAh cm^{-2} K metal: (a) Fe$_{SA}$-HCB, (b) HCB and (c) bare Al.

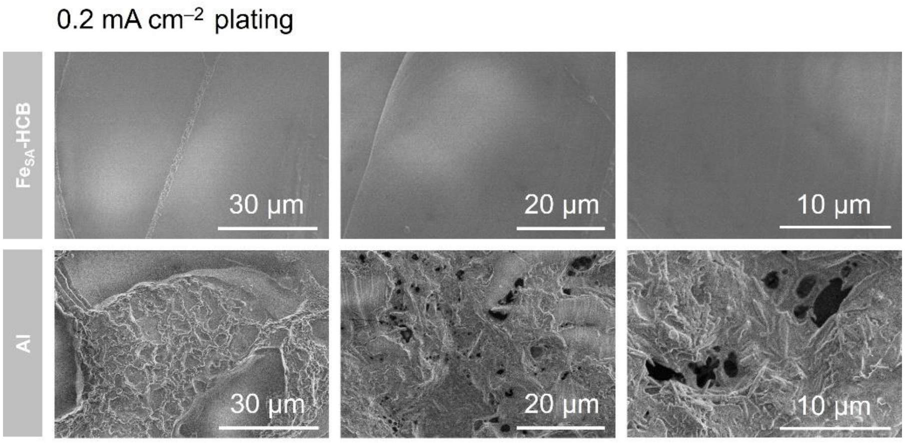

Fig. S27. SEM images of Fe_{SA}-HCB and Al current collectors after being plated with 0.2 mAh cm^{-2} of K metal.

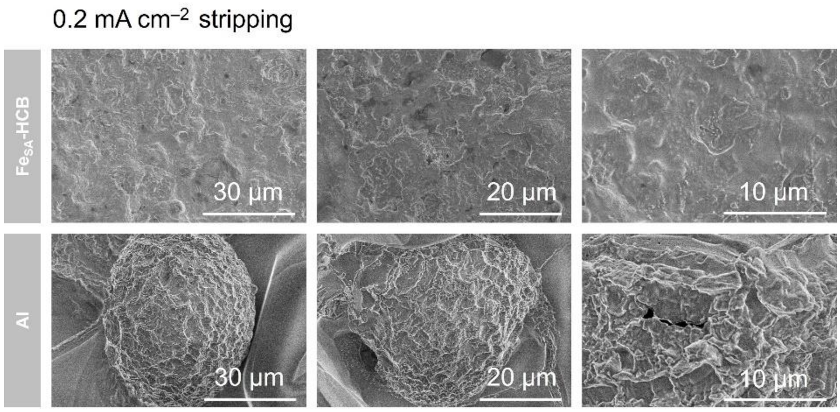

Fig. S28. SEM images of Fe_{SA}-HCB and Al current collectors after being stripped with 0.2 mAh cm^{-2} of K metal.

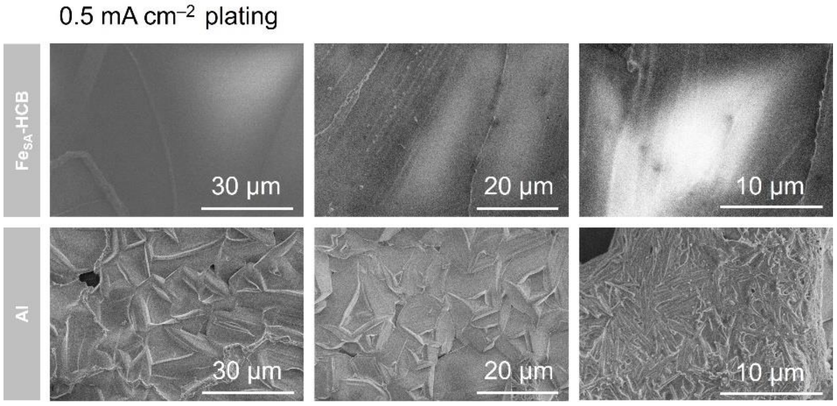

Fig. S29. SEM images of Fe_{SA}-HCB and Al current collectors after being plated with 0.5 mAh cm^{-2} of K metal.

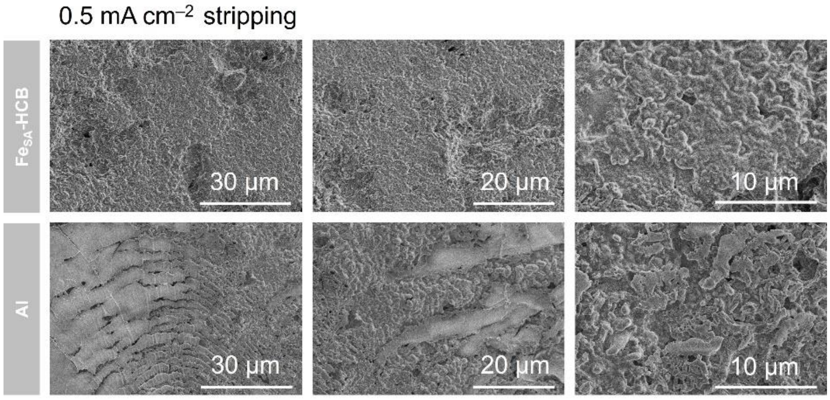

Fig. S30. SEM images of Fe_{SA}-HCB and Al current collectors after being stripped with 0.5 mAh cm^{-2} of K metal.

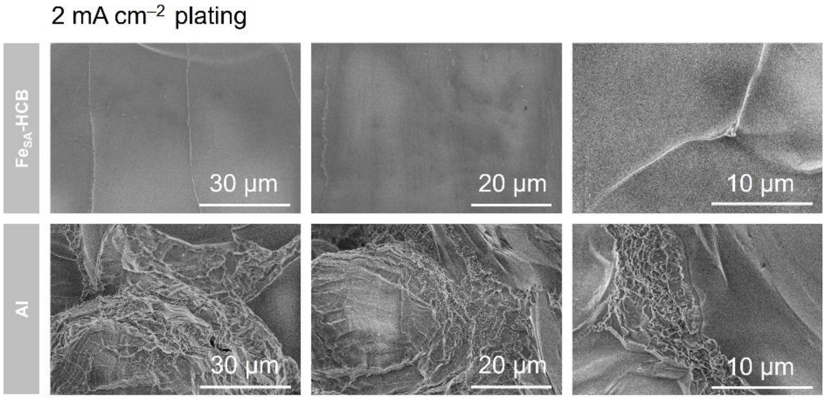

Fig. S31. SEM images of Fe_{SA}-HCB and Al current collectors after being plated with 2.0 mAh cm^{-2} of K metal.

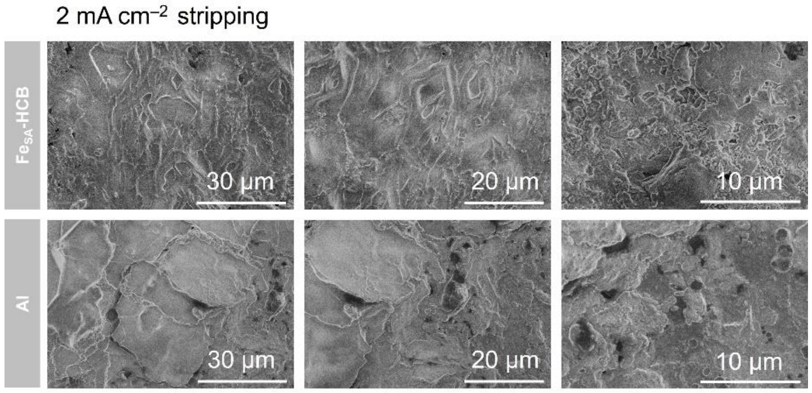

Fig. S32. SEM images of Fe_{SA}-HCB and Al current collectors after being stripped with 2.0 mAh cm^{-2} of K metal.

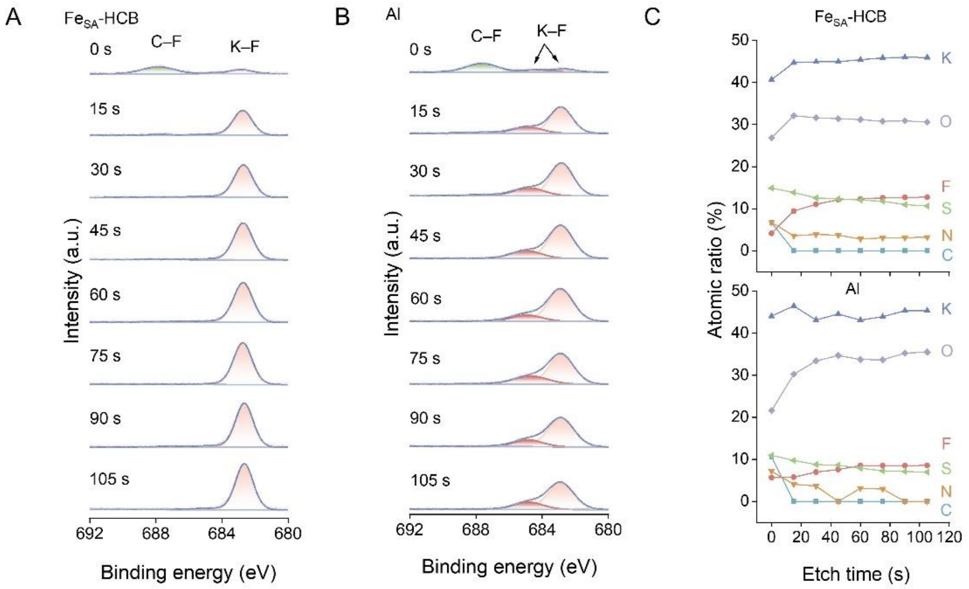

Fig. S33. XPS F 1*s* spectra at different sputtering times of (A) Fe_{SA}-HCB and (B) Al. (C) XPS quantitative analysis of the SEI derived from Fe_{SA}-HCB and Al with sputtering time from 0 to 105 s.

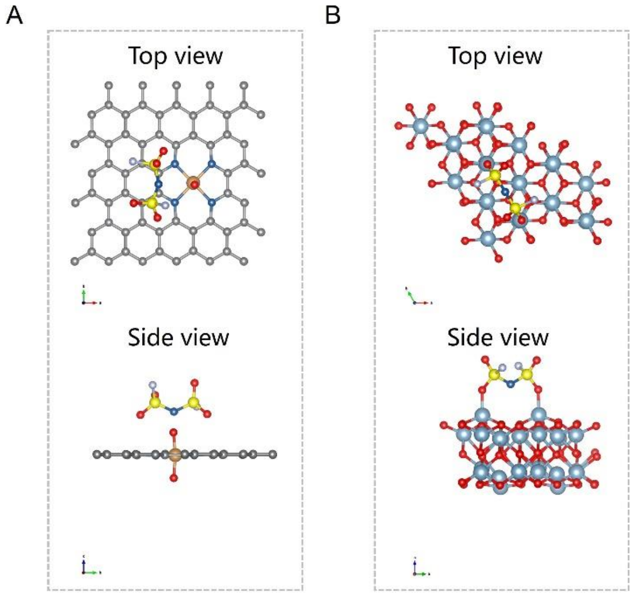

Fig. S34. The most stable configuration of FSI^- adsorbed on (A) Fe_{SA}-HCB and (B) Al_2O_3 substrate.

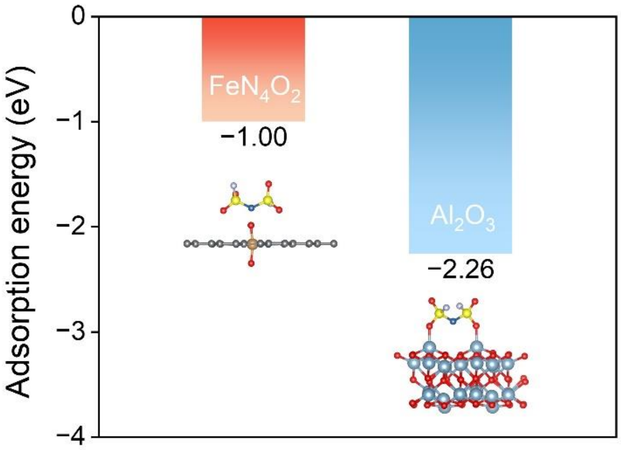

Fig. S35. The adsorption energy values of FSI⁻ adsorbed on the Fe_{SA}-HCB and Al_2O_3 substrate.

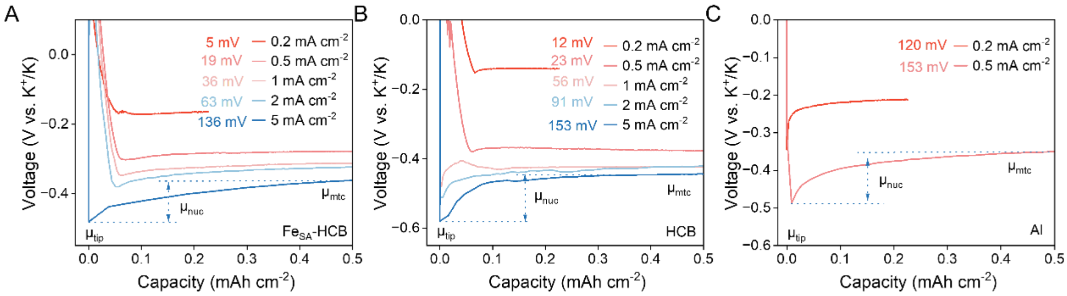

Fig. S36. Voltage profiles of K nucleation at different current density on (a) Fe_{SA}-HCB, (b) HCB and (c) bare Al.

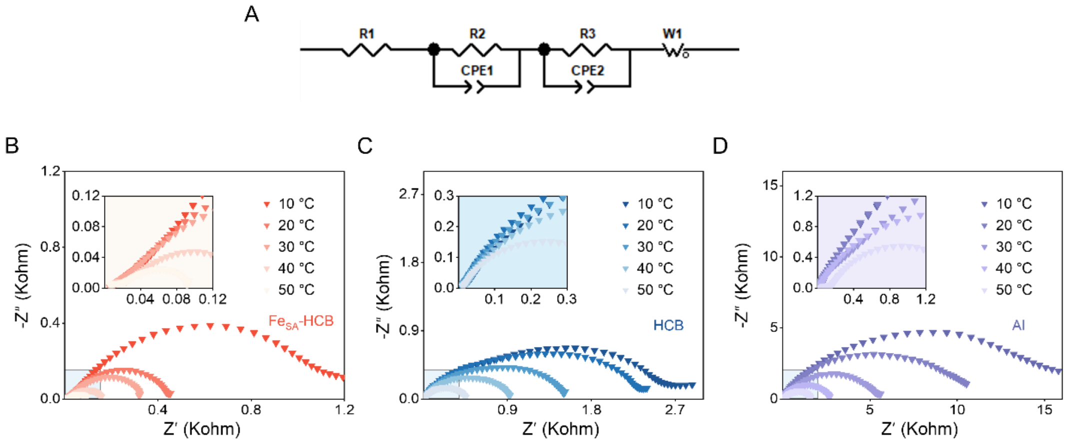

Fig. S37. (A) Equivalent circuit model used for fitting the impedance spectra. Temperature-dependent EIS curves for calculating activation energy of symmetric cells after 5 cycles with (B) Fe$_{SA}$-HCB, (C) HCB and (D) bare Al.

Notes: R_1 represents the internal resistance of the cell; R_2 and CPE_1 correspond to the SEI resistance and its non-ideal capacitive behavior; R_3 and CPE_2 represent the charge-transfer resistance and the associated double-layer capacitance; W_1 denotes the Warburg impedance related to ion diffusion.

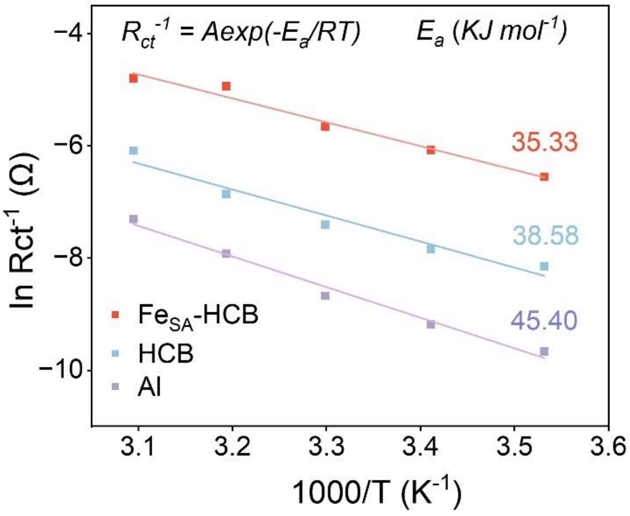

Fig. S38. Comparison of activation energies of Fe$_{SA}$-HCB, HCB and Al electrodes.

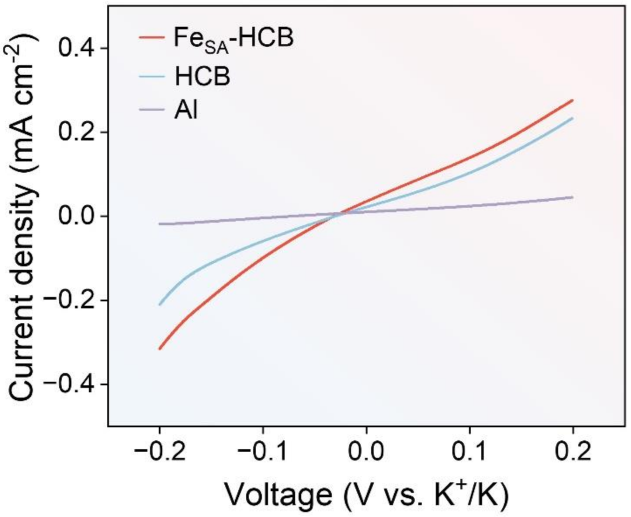

Fig. S39. LSV profiles of symmetric cells at a scan rate of 0.2 mV s^{-1}.

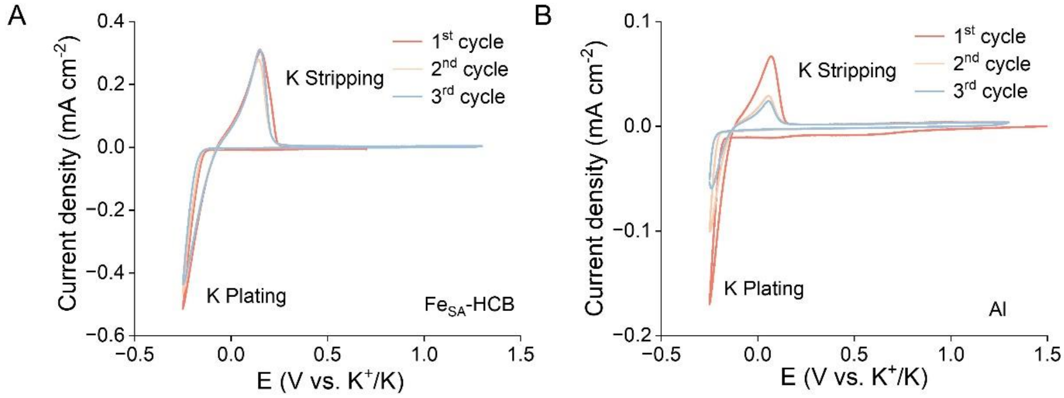

Fig. S40. CV curves of asymmetric cell for (A) Fe_{SA}-HCB and (B) Al electrode at a scan rate of 5.0 mV s^{-1}.

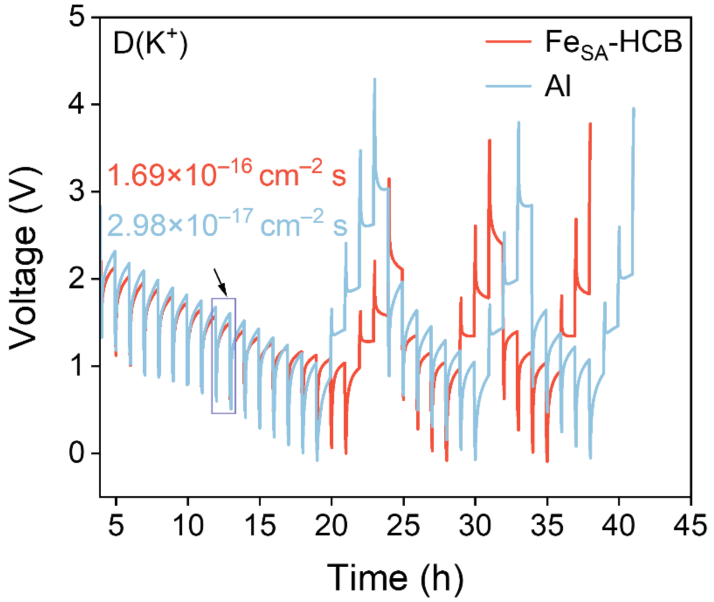

Fig. S41. Galvanostatic intermittent titration technique data showing the K^+ diffusion coefficients for Fe_{SA}-HCB and Al electrodes.

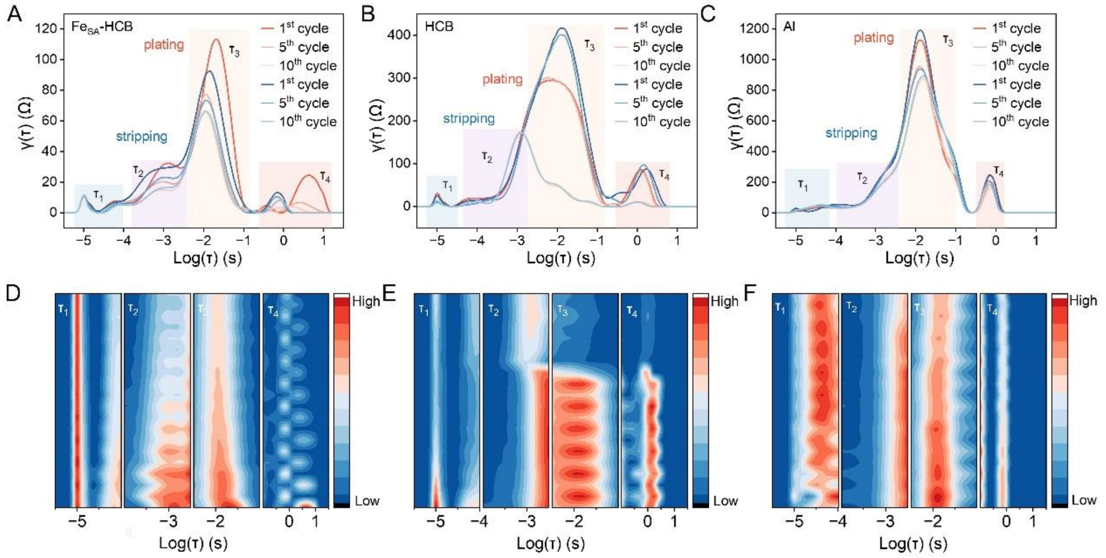

Fig. S42. (A), (B), (C) DRT analysis of deposition/stripping impedance (for 1st, 5th, 10th cycle) and (D), (E), (F) the corresponding mapping profiles for symmetric cells with Fe_{SA}-HCB, HCB and Al electrodes.

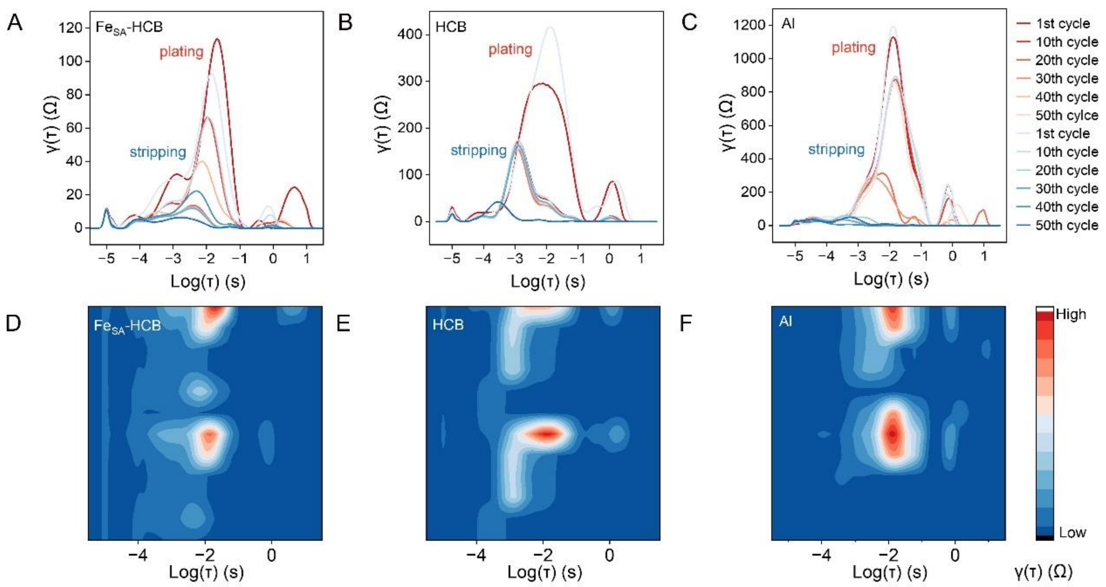

Fig. S43. (A), (B), (C) DRT analysis of deposition/stripping impedance (for 1st, 10th, 20th, 30th, 40th, 50th cycle) and (D), (E), (F) the corresponding mapping profiles (first 10 cycles) for symmetric cells with Fe_{SA}-HCB, HCB and Al electrodes.

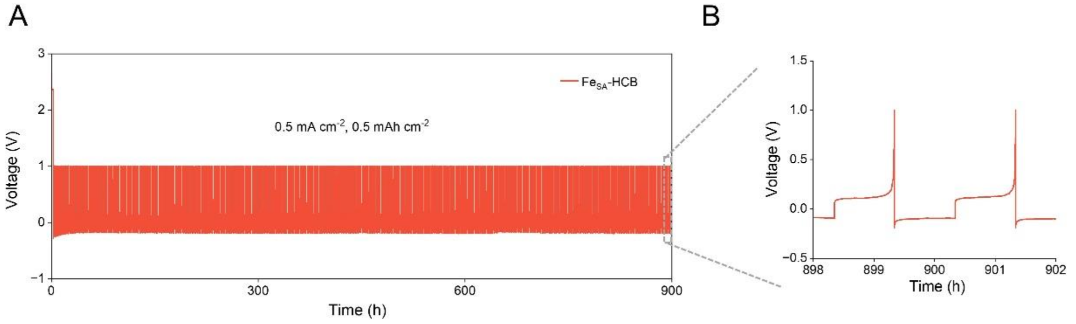

Fig. S44. (A) Voltage-time profiles of Fe_{SA}-HCB electrode at 0.5 mA cm^{-2}/0.5 mAh cm^{-2}. (B) Enlarged image in the gray box on the left.

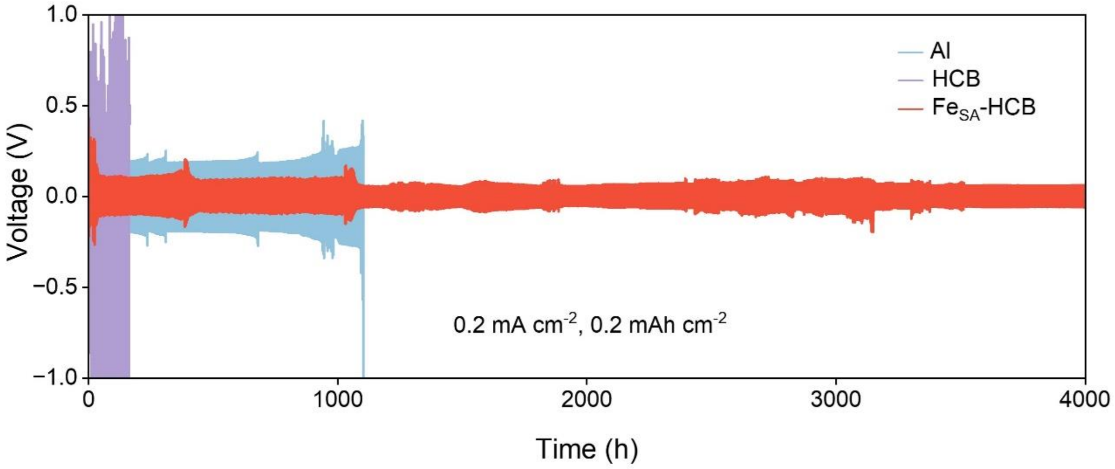

Fig. S45. Cyclic stability of symmetric cells at 0.2 mA cm^{-2}/0.2 mAh cm^{-2}.

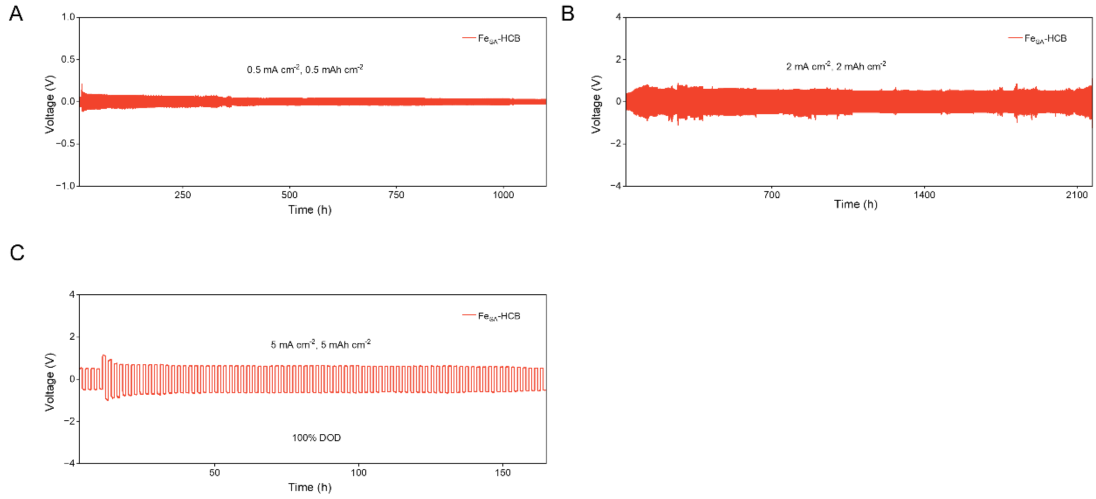

Fig. S46. Cycle stability of the symmetric cells at (A) 0.5 mA cm^{-2}/0.5 mAh cm^{-2}, (B) 2.0 mA cm^{-2}/2.0 mAh cm^{-2} and (C) 5.0 mA cm^{-2}/5.0 mAh cm^{-2}.

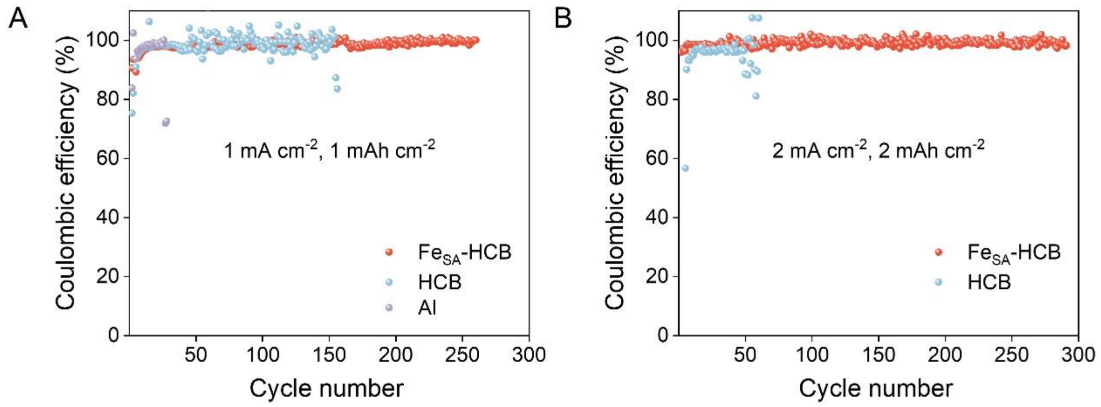

Fig. S47. Cycling stability of asymmetric cells based on different substrates. CE plots of K plating/stripping at (A) 1.0 mA cm^{-2} and (B) 2.0 mA cm^{-2}.

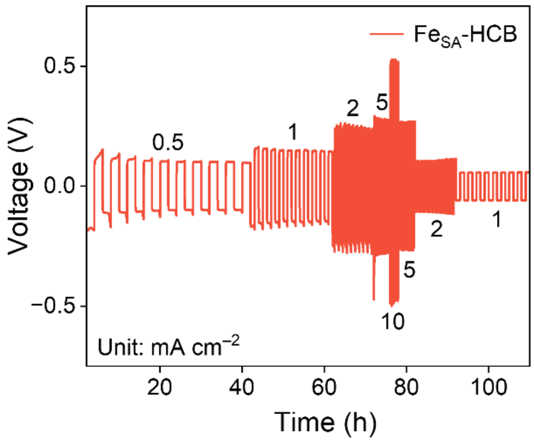

Fig. S48. Rate performance of Fe$_{SA}$–HCB||Fe$_{SA}$–HCB symmetric cells under progressively varied current densities at a fixed areal capacity of 1 mAh cm^{-2}.

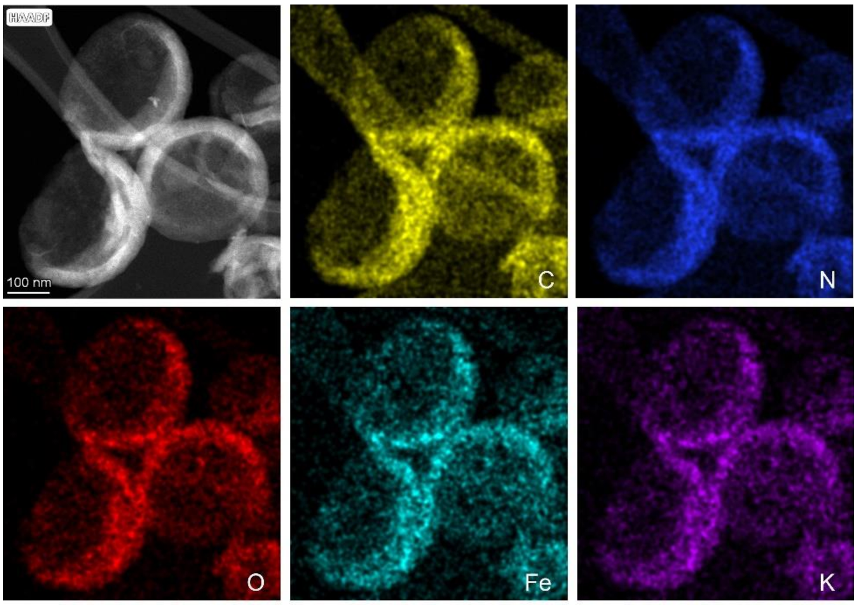

Fig. S49. HAADF-STEM image and corresponding element mappings of Fe_{SA}-HCB after cycling.

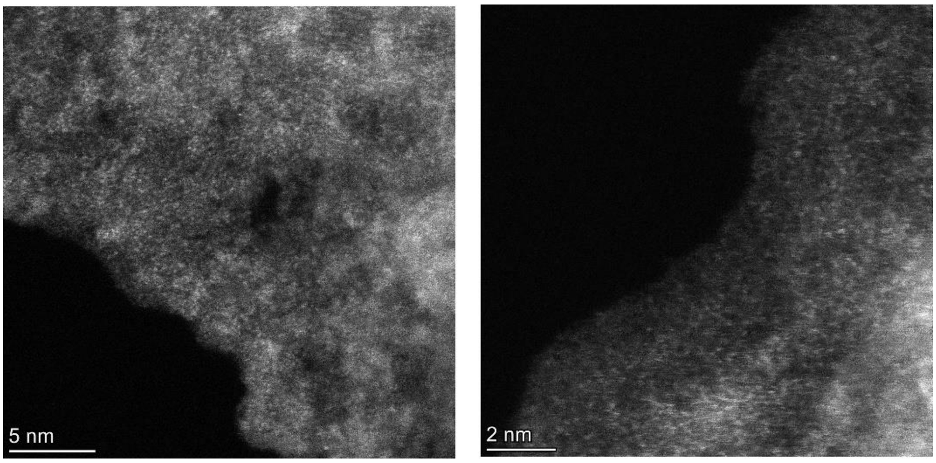

Fig. S50. AC-HAADF-STEM images of Fe_{SA}-HCB after cycling.

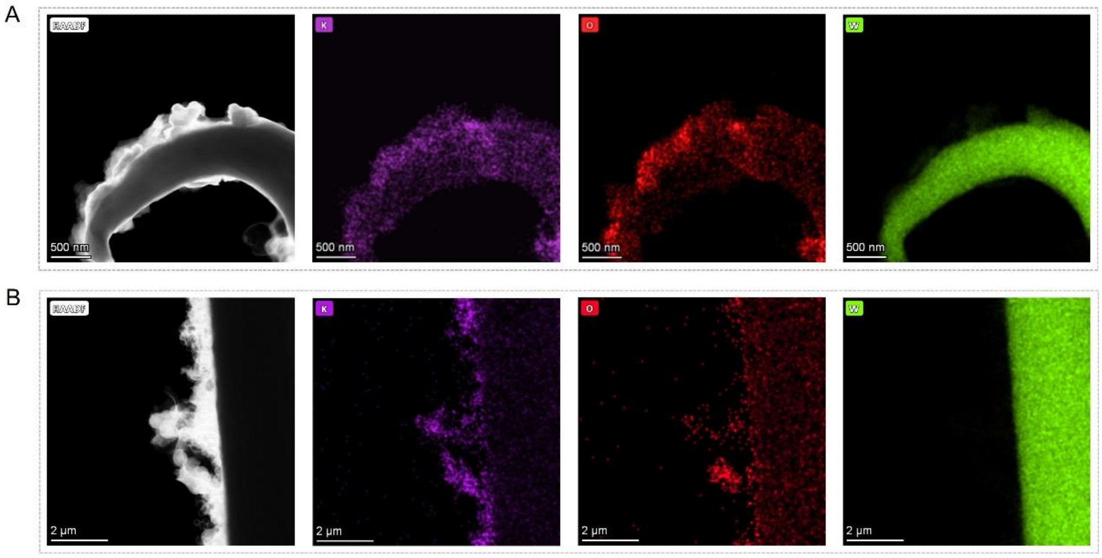

Fig. S51. HAADF-STEM image and K, O, and W element mappings of W tip.

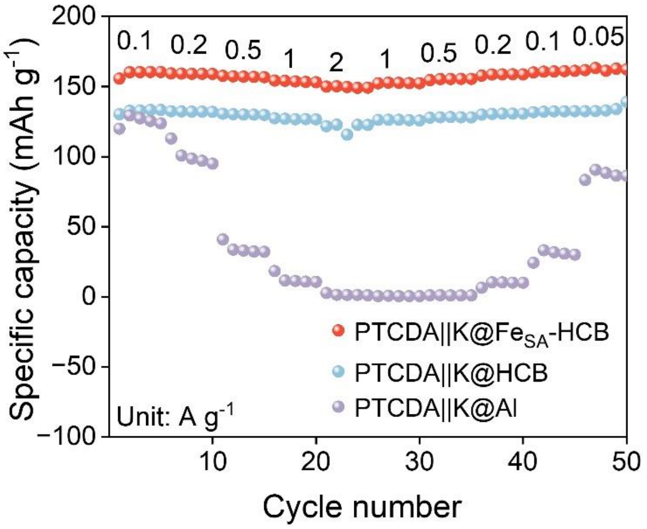

Fig. S52. Rate performance at 0.1, 0.2, 0.5, 1.0 and 2.0 A g^{-1}.

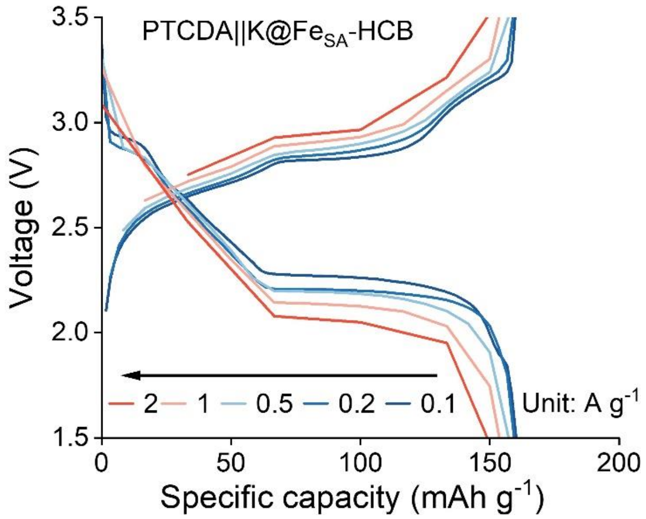

Fig. S53. GCD curves of PTCDA||K@Fe$_{SA}$-HCB at 0.1, 0.2, 0.5, 1.0 and 2.0 A g^{-1}.

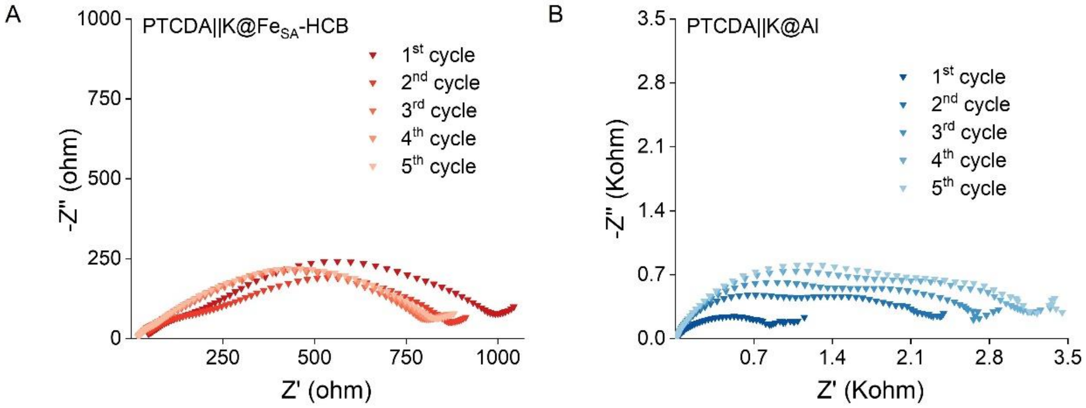

Fig. S54. Impedance of full cells with (A) Fe$_{SA}$-HCB and (B) Al electrode after 5 cycles.

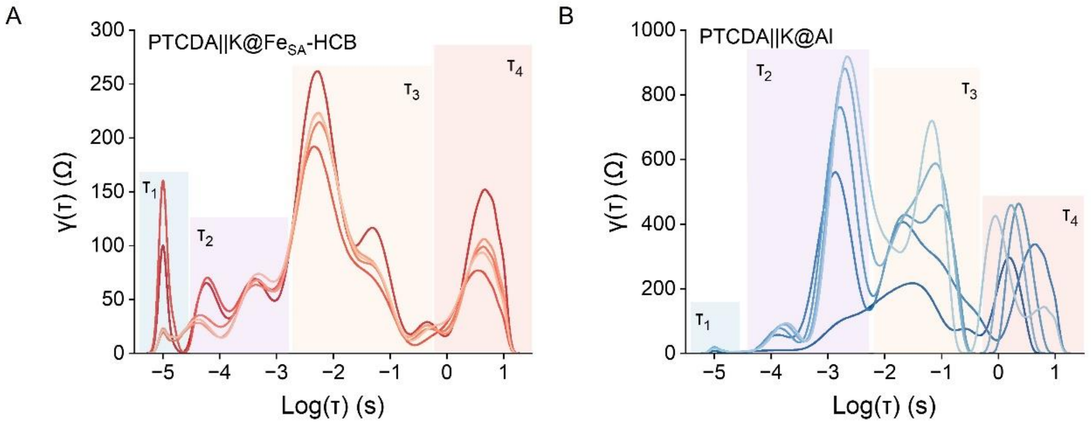

Fig. S55. DRT analysis of the impedance for full cells with (A) Fe$_{SA}$-HCB and (B) Al electrode after 5 cycles.

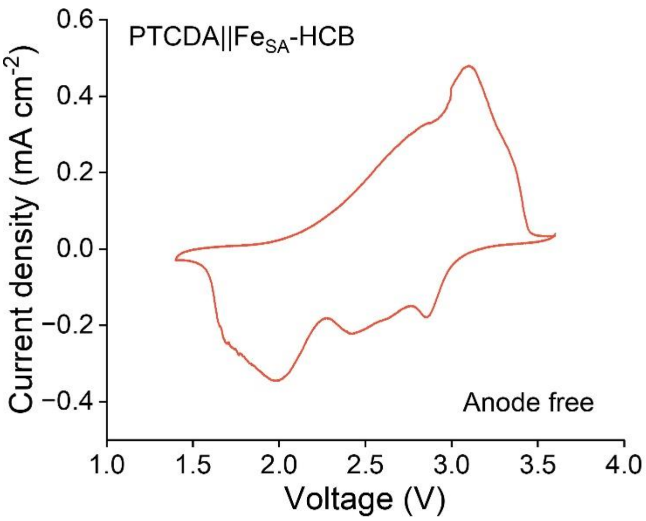

Fig. S56. CV profile at 1 mV s^{-1} for PTCDA||Fe$_{SA}$-HCB anode-free full cell.

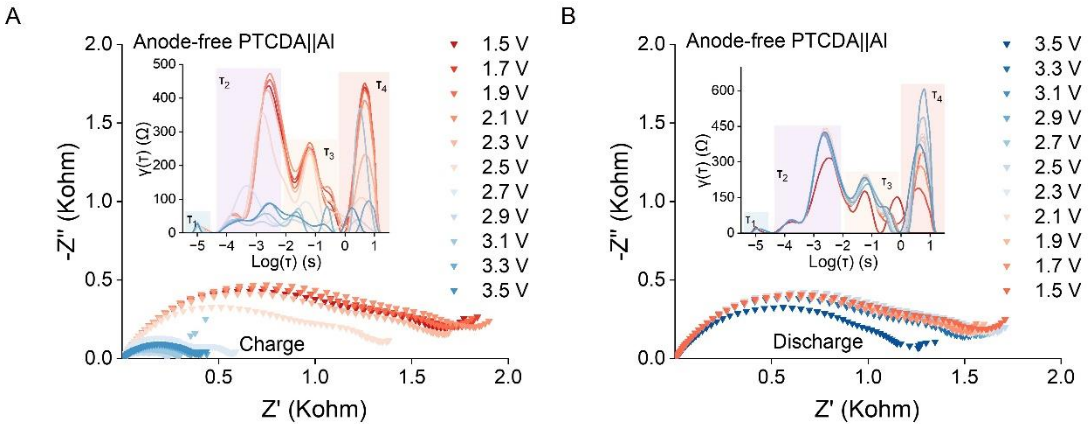

Fig. S57. *In situ* EIS and DRT deconvolution of the bare Al based anode-free full cell during (A) charging and (B) discharging.

Table S1. ICP-OES analysis results of the synthesized materials.

m_{HCB}: m_{FePc}	Material	Entry	Fe Content (wt%)	Etching
1: 2	Fe_{SA}-HCB	1	8.00%	0.75%
		2	8.00%	0.35%
		3	7.70%	0.50%
	Fe_{SA}-HCB-H_2O_2	1	2.40%	1.40%
		2	2.00%	1.30%
		3	3.40%	1.60%

Table S2. Raman data of samples fitted using a Gauss-Lorentz function.

Material		Peak type	Peak position	Intensity	FWHM	I_D/I_G
	0 mL H_2O_2	D	1337.6	1590.0	67.2	0.87
		G	1574.6	1825.5	61.1	
	0.5 mL H_2O_2	D	1334.4	1880.0	156.1	0.99
		G	1568.4	1891.0	89.9	
HCB	1.5 mL H_2O_2	D	1341.0	1355.8	84.4	0.98
		G	1576.2	1373.9	82.5	
	3.0 mL H_2O_2	D	1340.8	2075.6	75.3	1.08
		G	1569.9	1915.5	67.3	
	4.5 mL H_2O_2	D	1340.7	1947.2	72.7	1.08
		G	1577.6	1794.8	64.2	
	6.0 mL H_2O_2	D	1334.4	1184.2	92.7	1.05
		G	1576.1	1122.4	77.3	
	HCB-H_2O_2	D	1331.2	2335.5	84.3	1.01
		G	1574.5	2332.8	80.3	
	Fe_{SA}-HCB	D	1334.0	1921.1	82.3	1.04
		G	1576.0	1842.0	70.3	

Table S3. ICP-OES analysis results of the synthesized materials.

m_{HCB}: m_{FePc}	Amount of H_2O_2 (mL)	Entry	Fe Content after etching (wt%)
1: 2	0	1	1.4%
	0.5	2	1.3%
	1.5	3	1.7%
	3.0	4	1.7%
	4.5	5	1.4%
	6.0	6	1.7%
1: 1	1.5	1	3.5%
1: 2		2	1.7%
1: 3		3	0.7%

Table S4. C, N, O and Fe content from XPS analysis for the synthesized materials.

Material	C Content (wt%)	N Content (wt%)	O Content (wt%)	Fe Content (wt%)
Fe_{SA}-HCB	79.44	11.46	7.27	1.83
HCB-H_2O_2	80.2	11.78	8.02	/
HCB	92.52	12.75	7.06	/

Table S5. EXAFS fitting parameters at the Fe K-edge for various samples (S_0^2=0.76).

Sample	Shell	N[a]	R(Å)[b]	σ^2(Å^2)[c]	ΔE_0 (eV)[d]	R factor
Fe foil	Fe-Fe	8*	2.47±0.01	0.0052	7.0±1.0	0.0034
	Fe-Fe	6*	2.85±0.01	0.0065		
Fe_{SA}-HCB	Fe-N/O	5.9±0.2	2.02±0.01	0.0138	5.1±0.8	0.0043
	Fe-C	1.2±0.2	3.01±0.02	0.0002		

Notes: *a*: coordination numbers; *b*: bond distance; *c*: Debye-Waller factors; *d*: the inner potential correction. R factor: goodness of fit. S_0^2 was set to 0.76, according to the experimental EXAFS fit of Fe foil reference by fixing CN as the known crystallographic value.

Parameters of EXAFS fitting

The obtained XAFS data was processed in Athena (version 0.9.26) for background, pre-edge line and post-edge line calibrations. Then Fourier transformed fitting was carried out in Artemis (version 0.9.26). The k^2 weighting, k-range of 3 - 14 Å^{-1} and R range of 1 - 3 Å were used for the fitting of Fe foil; k-range of 3 – 10.2 Å^{-1} and R range of 1 - 3 Å were used for the fitting of samples. The four parameters, coordination number, bond length, Debye-Waller factor and E0 shift (CN, R, ΔE_0) were fitted without anyone was fixed, the σ^2 was set.

Parameters of Wavelet Transform

For Wavelet Transform analysis, the $\chi(k)$ exported from Athena was imported into the Hama Fortran code. The parameters were listed as follow: R range, 1 - 4 Å, k range, 0 - 12 Å^{-1} for samples; k weight, 2; and Morlet function with κ=10, σ=1 was used as the mother wavelet to provide the overall distribution.

Table S6. Quantitative comparison of average voltage hysteresis (mV) in symmetric cells at 0.2 mA cm^{-2}.

Material \ Cycle	1st	50th	100th	1000th
Fe_{SA}-HCB	157	109	100	50
Al	500	300	207	/

Table S7. Benchmarking comparison between this work and anode-free alkali-metal batteries reported in recent literatures.

CC material	Cathode	Current density (mA g^{-1})	Cycle number	Capacity retention (%)	Ref.
Fe_{SA}-HCB	PTCDA	200	200	81.2	**This work**
Al@G	FeS_2	100	30	40	*Zhao et al. (8)*
Cu_6Sn_5@Cu	PTCDA	20	30	69.4	*Ren et al. (14)*
Cu	NFM	200	150	82.3	*Xing et al. (64)*
Ni	NFM	200	100	45.4	*He et al. (65)*
CeF_3@NC	NVP	200	100	83.6	*Xu et al. (66)*
CNF@Al	MnHCF	100	50	70	*Yu et al. (70)*
Al	PTPAn	200	300	62.5	*Huang et al. (69)*
Cu	PTCDA	200	50	82	*Tang et al. (68)*
C@Al	NVP	110	100	70	*Chen et al. (67)*
